# Impact of the Season on Total Polyphenol and Antioxidant Properties of Tea Cultivars of Industrial Importance in Northeast India

**DOI:** 10.3390/foods12173196

**Published:** 2023-08-24

**Authors:** Himangshu Deka, Podma Pollov Sarmah, Pritom Chowdhury, Kaberi Rajkhowa, Santanu Sabhapondit, Saumik Panja, Tanmoy Karak

**Affiliations:** 1 Biochemistry Department, Tocklai Tea Research Institute, Jorhat 785008, Assam, India; p.pollov@tocklai.net (P.P.S.); krajkhowa1996@gmail.com (K.R.); santanusabhapondit1@gmail.com (S.S.); 2 Biotechnology Department, Tocklai Tea Research Institute, Jorhat 785008, Assam, India; pritomc@gmail.com; 3 Environment, Health and Safety, University of California, San Francisco 505 Parnassus Ave, San Francisco, CA 94143, USA; saumik.panja@ucsf.edu; 4 Department of Soil Science, School of Agricultural Sciences, Nagaland University, Medziphema Campus, Medziphema 797106, Nagaland, India

**Keywords:** antioxidant activity, harvesting season, Tocklai vegetative (TV) cultivar, total polyphenol

## Abstract

Tocklai vegetative (TV) cultivars are extensively planted in the tea-growing regions of Northeast India. The present investigation explores the impact of season on the total polyphenol (TP) content and the antioxidant activity of thirty-one TV cultivars (TV1–TV31) and four other commercially popular cultivars, namely, Betjan, Kharijan, S.3A/3, and T.3E/3. The TP content of the cultivars was observed to be highest in the monsoon season, with values ranging from 230.57 to 283.53 mg g^−1^. In the pre-monsoon season and autumn, the TP content ranged from 197.87 to 256.77 mg g^−1^ and from 169.97 to 223.50 mg g^−1^, respectively. Antioxidant activity was measured through DPPH, ABTS, FRAP, and lipid peroxidation inhibition assays. The cultivars showed the highest antioxidant activity in the monsoon in tandem with TP content. A bivariate correlation indicated a highly significant (*p* ≤ 0.01) positive correlation of antioxidant activity with TP content (R^2^ = 0.83–0.96).

## 1. Introduction

Tea is a popular beverage consumed worldwide for its refreshing taste and health-beneficial properties. Processed teas are broadly classified into white, green, oolong, black, and dark tea based on the liquor character [1], which differs due to the variation in processing, and degree of fermentation during manufacturing of different tea types. Various phytochemicals like polyphenols, purine alkaloids, amino acids, organic acids, carotenoids, etc. are present in tea (*Camellia sinensis* (L.) O. Kuntze), to which its health properties are mainly attributed. One major benefit of these molecules, particularly polyphenolic compounds, is their antioxidant activity, which scavenges reactive oxygen species (ROS) produced inside our body. ROS are generated in our body as a cellular metabolic byproduct that plays the role of secondary messengers that influence various physiological functions of the body [2]. Many cell line and animal model studies have reported the health benefits of tea against several diseases such as diabetes, cancer, cardiovascular diseases, etc. [3,4,5,6]. Green tea contains various polyphenolic classes of compounds like catechins (flavan-3-ols), flavonols, phenolic acids, anthocyanins, etc., of which catechins are the major constituents, contributing up to 80% to the total polyphenol (TP) content and specifically found in tea to that extent [7,8]. The major catechins found in green tea are (−) epigallocatechin-3-gallate (EGCG), (−) epigallocatechin (EGC), (−) epicatechin-3-gallate (ECG), (−) epicatechin (EC), and (+) catechin (+C). In black tea, these catechins get oxidized and polymerized during fermentation to form the black tea polyphenolic class of compounds, theaflavins and thearubigins. The flavonol content in tea varies from 1% to 2% and is primarily found in the glycosylated form as mono-, di-, and tri-glycosides. Aglycones such as kaempferol, myricetin, and quercetin are also present in tea in small quantities [1,9]. Flavonols including kaempferol-3-*O*-rutinoside, kaempferol-3-*O*-glucoside, quercetin-3-*O*-rutinoside, quercetin-3-*O*-glucoside, quercetin-3-*O*-galactoside, and myricetin-3-*O*-galactoside are the major contributor to the astringency of tea [10]. Phenolic acids are another class of polyphenols in tea, and it accounts for around 5% of the dry weight of fresh leaves [10]. Phenolic acids in tea primarily include theogallin, gallic acid, caffeic acid, and chlorogenic acid [1,10]. These acids impart a sour and astringent taste to tea infusion [10]. Anthocyanins are one of the intense colored pigments present in tea having antioxidative properties [11]. This class of compounds includes cyanidin, delphinidin, malvidin, pelargonidin, peonidin, cyanidin-3-*O*-galactoside, pelargonidin-3,5-diglucoside, cyanidin-3-*O*-glucoside, etc. [11,12,13]. Apart from the antioxidant activity, TP is also a quality indicator of tea [14]. TP contributes to sensory properties like the astringency in green tea [15], whereas the unoxidized TP in black tea imparts mouth fulness and astringency [16]. The content of these TP varies greatly among various tea cultivars used for manufacturing teas. The variation in TP also depends on the harvesting season as secondary metabolites significantly increase in summer compared to the other seasons due to the increase in temperature and total sunlight [7]. Thus, the antioxidant activity of tea, which has a positive correlation with TP [17], also depends on the type of cultivar, harvesting season, and processing method [18].

Tocklai Tea Research Institute (TTRI), Assam, India, has developed 35 vegetatively propagated tea cultivars that are widely used by the tea industry of India. In a previous study, we reported seasonal variations of catechins and caffeine content in 31 out of 35 tea cultivars developed by TTRI and 4 other commercially popular cultivars. Zhang et al. reported the variation in antioxidant activity in terms of the changes in the geographical origin, plantation elevation, and leaf grade [19]. Carloni et al. reported variations in antioxidant activity in different types of tea processed from the same batch of fresh leaves collected from the same cultivar [20]. A group of Kenyan researchers studied the antioxidant activity of different types of processed tea of the same geographical origin but collected from different tea factories in Kenya [21]. Saito et al. studied the antioxidant activity of green tea processed from the leaves of a single cultivar in different months of harvesting seasons [22]. However, no study has been carried out so far on the seasonal variation in TP and antioxidant activity in tea. In continuation of our earlier work, here, we report the variation in TP and antioxidant activity of tea concerning the harvesting season in 31 tea cultivars (TV1 to TV31) developed by TTRI and 4 commercially popular cultivars (Betjan, Kharijan, S.3A/3, and T.3E/3) from the Northeast region of India. A statistical correlation of TP content and antioxidant activity with the principal catechins (+C, EC, EGC, EGCG, and ECG) and caffeine content of the cultivar, already reported in one of our earlier publications [7], is also drawn.

## 2. Materials and Methods

### 2.1. Chemicals

2,2′-azinobis (3-ethylbenzothiazoline-6-sulfonic acid) diammonium salt (ABTS), 2,2-diphenyl-1-picrylhydrazyl (DPPH), 2,4,6-tri(2-pyridyl)-s-triazine (TPTZ), 6-hydroxy-2,5,7,8-tetramethylchroman-2-carboxylic acid (Trolox), potassium persulfate, gallic acid monohydrate (≥98.0%), iron (III) chloride hexahydrate, tween 20, fish oil, and trichloroacetic acid (TCA) were purchased from Sigma-Aldrich, Steinheim, Germany. 2-thiobarbituric acid (TBA), butylated hydroxytoluene (BHT), and Folin & Ciocalteu’s phenol (FCP) reagent were purchased from HiMedia, Nashik, India. Acetic acid, sodium acetate trihydrate, iron (II) sulphate heptahydrate, hydrochloric acid, and sodium carbonate (anhydrous) were purchased from Merck, Mumbai, India.

### 2.2. Instrumentation

A UV-Vis spectrophotometer (make: Varian, New South Wales, Australia; Model: Cary 50) was used for measuring the absorbance of different reaction mixtures. The water used in all experiments was purified using a water purification system (Make: Merck Millipore, Darmstadt, Germany). The pH measurements and adjustments were performed by using a pH meter (Eutech Instruments, Mumbai, India; Model: pH 510). A high-speed centrifuge 6–16K from Sigma, Germany was used for centrifuging the tea extracts.

### 2.3. Tea Leaf Sampling

Young leaf samples from cultivars TV1–TV31 and four other commercially popular cultivars, namely, Betjan, Kharijan, S.3A/3, and T.3E/3, were collected from Borbheta Experimental Tea Estate of TTRI. The tea estate is located between 26°43′14″ N and 94°11′54″ E, with an average elevation of 96.5 m above mean sea level and an average precipitation of 2036 mm (Supplementary information, Figure S1). These cultivars include various varieties such as Assam (16), Cambod (14), Assam hybrid (2), Assam China hybrid (2), and China hybrid (1). The origin and year of release of these cultivars are available in our earlier work [7]. The growing environment, soil nutrient management, and age of the plants (30–35 years) were maintained identically for the experimental study. The buds along with the first two leaves were hand plucked in three harvesting seasons viz the pre-monsoon season (April and May), the monsoon season (July and August), and autumn (October and November) of 2017, with three biological replications in every season. The fresh tea leaf samples were enzyme-deactivated using steam for 90 s followed by drying in a hot air oven at 60 ± 2 °C. The samples were powdered and sieved using a 250-micron sieve and then stored at −80 °C until further analysis.

### 2.4. Determination of Total Polyphenol

The TP content was determined using the Folin–Ciocalteu method developed by the International Organization for Standardization [23]. Briefly, 0.200 g of finely powdered tea samples were extracted twice with 5 mL of 70% methanol at 70 °C in a water bath. The extraction volume was made up to 10 mL with the extraction solvent. The extracts were then diluted 100 times before the estimation of TP. The diluted extracts (1 mL) were allowed to react with the FCP reagent (5 mL, 10% solution) in an alkaline medium created by adding 4 mL of a sodium carbonate solution (7.5%, *w*/*v*). The absorbance of the reaction mixture was measured at 765 nm using a spectrophotometer after being allowed to stand for 1 h at room temperature. A sample blank was also prepared using water instead of diluted tea extract. The TP content was determined against a gallic acid calibration curve (y = 0.0121x − 0.002, R^2^ = 0.999) prepared using varying concentrations (10–50 µg mL^−1^).

### 2.5. Preparation of Tea Extracts for Antioxidant Activity Study

The extraction of tea samples was carried out as described by Deka et al. [24] with little modification: 2.5 g of finely ground tea sample was extracted with 25 mL of water in boiling condition for 3 min. The extracts were allowed to cool and centrifuged at 12,000 rpm for 10 min for the removal of suspended matter. The supernatant was collected into a 25 mL volumetric flask, and the remainder of the flask was filled with water. The extracts thus prepared were used for antioxidant activity measurements.

### 2.6. DPPH Radical Scavenging Activity

A DPPH assay was performed using the method reported by Niknam et al. with slight modification [25]. Briefly, 2.5 mL of a 1 mM ethanolic solution of DPPH was mixed with 0.25 mL of the tea extract and further diluted with 2.5 mL of 70% ethanol to make a total volume of 5.25 mL. The mixture was shaken vigorously using a vortex mixer and left for 30 min at room temperature in dark conditions. The absorbance was read at 517 nm in a spectrophotometer. A blank sample was prepared using 70% ethanol (0.25 mL) instead of the tea extract. The DPPH scavenging activity of the tea extract was calculated using the following formula:

DPPH activity (%) = [(A_B_ − A_E_)/A_B_] × 100 (1)

where A_B_ and A_E_ are the absorbances of the blank sample and reaction mixture, respectively. A calibration curve with varying concentrations of Trolox (0.10–0.40 mM) was also prepared, and the results were expressed in mM Trolox equivalent (TE) per gram of tea.

### 2.7. ABTS Radical Scavenging Activity

The free radical scavenging activity of tea extracts was also studied using the ABTS radical cation decolorization assay [26]. The ABTS radical cation (ABTS^•+^) was generated by reacting a 7 mM aqueous ABTS solution with 2.45 mM potassium persulfate (K_2_S_2_O_8_) in dark conditions at room temperature for 12–16 h. Before use, the ABTS^•+^ solution was diluted 50-fold with water to obtain an absorbance in the range from 0.3 to 0.8 at 734 nm. All reagents were prepared freshly before analysis. An aliquot of 200 μL of the tea extract (400 fold diluted) was added to 6 mL of a diluted ABTS^•+^ solution and then the reaction mixture was allowed to stand in the dark for 1 h. A blank sample was prepared using 200 μL of water instead of the tea extract. The absorbance was read at 734 nm in a spectrophotometer. The scavenging activity was calculated using the following formula:

ABTS activity (%) = [(A_B_ − A_E_)/A_B_] × 100 (2)

where A_B_ and A_E_ are the absorbances of the blank sample and reaction mixture, respectively. A calibration curve with varying concentrations of Trolox (0.20–0.70 mM) was also prepared, and the results were expressed in mM TE per gram of tea.

### 2.8. Ferric-Reducing Antioxidant Potential (FRAP) Assay

The ferric-reducing potential of tea extracts was measured using the method of Benzie and Strain with minor modifications [27]. This approach is based on the reduction of colorless Fe^3+^-tripyridyltriazine to a blue-colored Fe^2+^-tripyridyltriazine complex at low pH via the phenolic compounds of tea. A working reagent was prepared by mixing a 300 mM acetate buffer (pH 3.6), 10 mM TPTZ (2,4,6-tri(2-pyridyl)-s-triazine) in 40 mM hydrochloric acid, and 20 mM ferric chloride in a volume ratio of 10:1:1. All reagents were prepared freshly before analysis. In total, 0.5 mL of the tea extract (1000 fold diluted) was added to 7 mL of the working reagent, and the reaction mixture was incubated at 37 °C for 30 min in a water bath. The color development was measured at 593 nm in a spectrophotometer. A blank reading was taken using an acetate buffer instead of the tea extract. The difference in absorption between the sample reaction mixture and the blank was used to calculate the FRAP. A calibration curve was prepared using different concentrations (0.10–0.80 mM) of FeSO_4_.7H_2_O. FRAP values were expressed as mM Fe^2+^ per gram of tea sample.

### 2.9. Lipid Peroxidation Inhibition Assay

An oil emulsion was prepared using the method of An et al. with minor modifications [28]. This oil emulsion serves as the system for lipid peroxidation. The oil emulsion was prepared by mixing 1 mL of fish oil, 10 mL of sodium phosphate buffer (0.1 M), and 0.5 mL of tween 20. The mixture was homogenized for 10 min, followed by the addition of 0.2 g of potassium hydroxide. The pH of the mixture was adjusted to 6.5 using 1 N HCl. The tea extract (0.5 mL) was mixed with the oil emulsion (0.5 mL) and incubated at 37 °C in a water bath for 1 h, and 0.5 mL of 5% BHT was added to the reaction mixture and mixed in a vortex mixer to stop the reaction. To this, 2 mL of a 20 mM TBA solution (prepared in 15% TCA) was added, and the mixture was incubated for 15 min in a boiling water bath. To the reaction mixture, 5 mL of *iso*-butanol was added and centrifuged for 15 min. The absorbance of the butanol layer was read at 531 nm in a spectrophotometer. A sample blank (B1) and an oil emulsion blank (B2) were prepared using 0.5 mL of millipore water in place of the tea extract and oil emulsion, respectively. The inhibition activity was calculated using the following formula:

Lipid peroxidation inhibition (%) = [1 − {(T1 − B2)/B1}] × 100 (3)

where T1 denotes the absorbance of the reaction mixture with a tea infusion.

### 2.10. Statistical Analysis

The data were presented as mean ± standard error (SE) of three independent measurements. One-way analysis of variance (ANOVA) of the generated data was performed using SPSS software version 17.00 (SPSS Inc., Chicago, IL, USA). The mean data of each parameter for each cultivar were differentiated using Tukey’s multiple comparison tests, and differences were considered significant at *p* ≤ 0.01 and *p* ≤ 0.05. The mean data of different parameters of the cultivars were compared using WASP–Web Agri Stat Package 2.0 at https://ccari.icar.gov.in/wasp2.0/index.php (accessed on 10 May 2023). Pearson correlations among the different biochemical parameters and antioxidant activity were generated using Origin Pro 2019b in the form of a heatmap to understand the association among them.

## 3. Results and Discussion

### 3.1. Total Polyphenol Content

The TP content of the cultivars in three seasons—the pre-monsoon, the monsoon, and autumn—was measured from harvested green leaves and is presented in Table 1.

**Figure 1:**
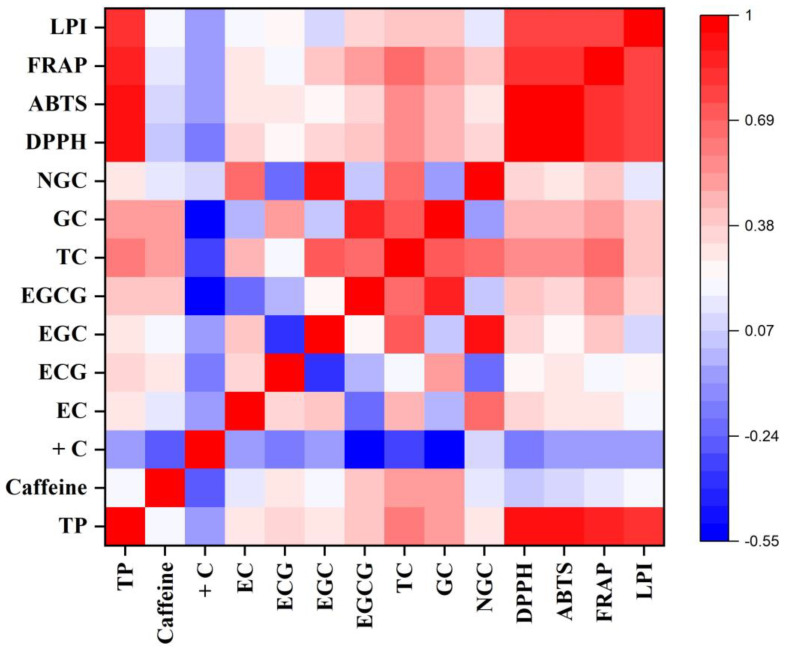
Heatmap of a correlation matrix between biochemical constituents and antioxidant activity in the pre-monsoon season. TP: total polyphenol; +C: (+)-catechin; EC: (−)-epicatechin; ECG: (−)-epicatechin-3-gallate; EGC: (−)-epigallocatechin; EGCG: (−)-epigallocatechin-3-gallate; TC: total catechin; GC: galloylated catechins; NGC: non-galloylated catechins; DPPH: DPPH radical scavenging activity; ABTS: ABTS radical scavenging activity; FRAP: ferric-reducing antioxidant potential; LPI: lipid peroxidation inhibition activity.

In the pre-monsoon season, the TP content varied from 197.87 ± 6.19 mg g^−1^ in TV8 to 256.77 ± 2.28 mg g^−1^ in TV3. A 30% difference was observed between the cultivars with the highest and lowest TP contents. The correlation study between TP and all major catechins except +C showed significant positive correlations (*p* ≤ 0.01) (Figure 1).

**Figure 2:**
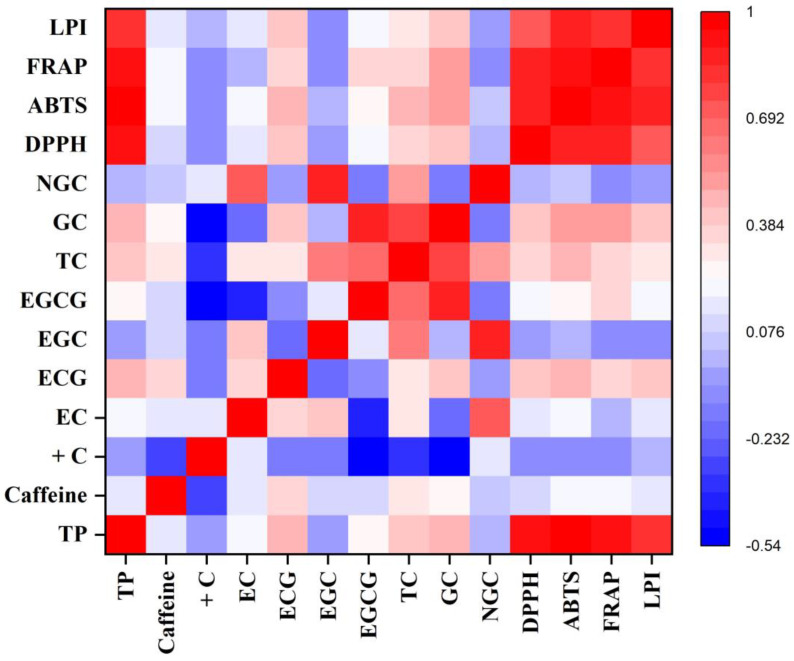
Heatmap of a correlation matrix between biochemical constituents and antioxidant activity in the monsoon season. TP: total polyphenol; +C: (+)-catechin; EC: (−)-epicatechin; ECG: (−)-epicatechin-3-gallate; EGC: (−)-epigallocatechin; EGCG: (−)-epigallocatechin-3-gallate; TC: total catechin; GC: galloylated catechins; NGC: non-galloylated catechins; DPPH: DPPH radical scavenging activity; ABTS: ABTS radical scavenging activity; FRAP: ferric-reducing antioxidant potential; LPI: lipid peroxidation inhibition activity.

In the monsoon season, the highest TP content was found in TV1 (283.53 ± 3.91 mg g^−1^), whereas TV21 showed the lowest value (230.57 ± 4.72 mg g^−1^), with 23% more TP content in TV1 in comparison with TV21. The TP content showed a significant positive correlation (*p* ≤ 0.01) with ECG and TC (TC: total catechin, which is a sum of the five major catechins) (Figure 2).

**Figure 3:**
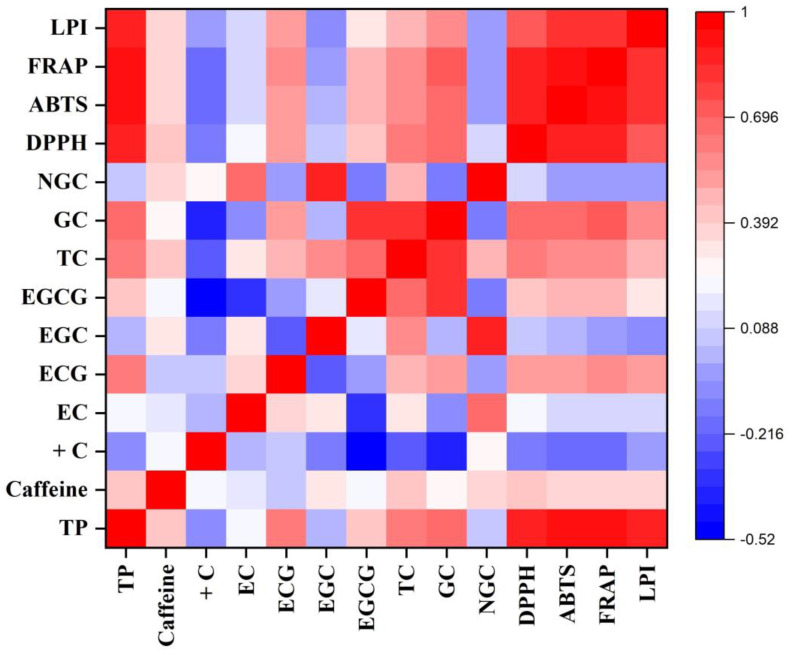
Heatmap of a correlation matrix between biochemical constituents and antioxidant activity in autumn. TP: total polyphenol; +C: (+)-catechin; EC: (−)-epicatechin; ECG: (−)-epicatechin-3-gallate; EGC: (−)-epigallocatechin; EGCG: (−)-epigallocatechin-3-gallate; TC: total catechin; GC: galloylated catechins; NGC: non-galloylated catechins; DPPH: DPPH radical scavenging activity; ABTS: ABTS radical scavenging activity; FRAP: ferric-reducing antioxidant potential; LPI: lipid peroxidation inhibition activity.

In autumn, TV9 had the highest TP content (223.50 ± 4.39 mg g^−1^), whereas TV27 had the lowest content (169.97 ± 1.82 mg g^−1^). This depicted a 31.5% variation in the TP content between the two cultivars with the highest and lowest values. Significant positive correlations (*p* ≤ 0.01) were observed for TP with EGCG, ECG, and TC (Figure 3).

**Figure 4:**
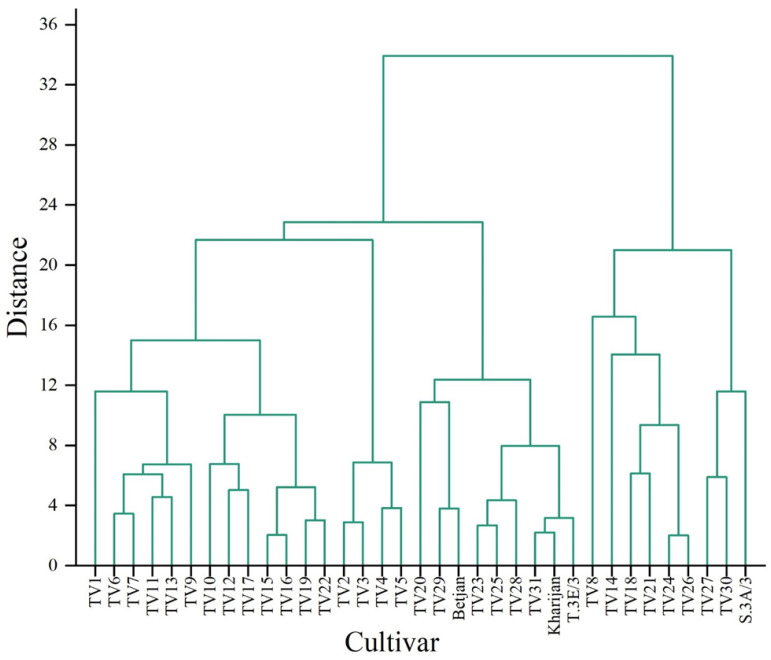
Dendrogram representing clustering of cultivars based on total polyphenol content of cultivars in the pre-monsoon season.

**Figure 5:**
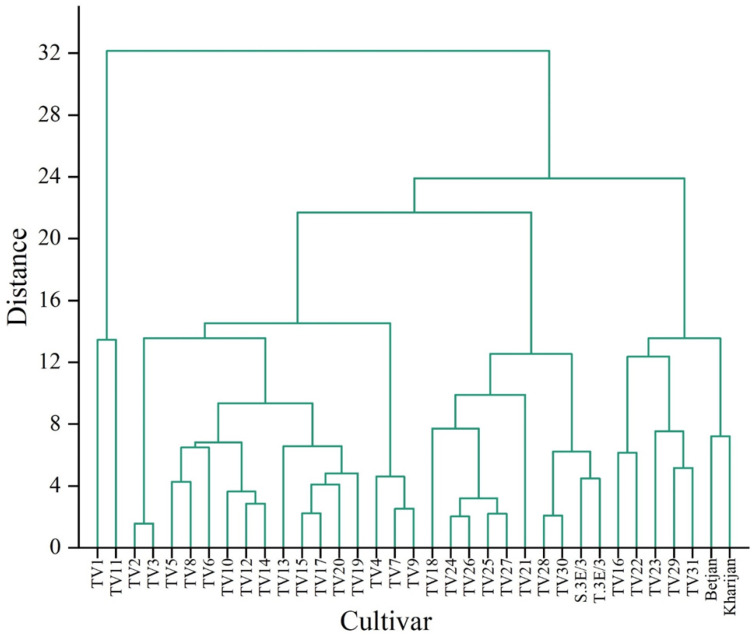
Dendrogram representing clustering of cultivars based on total polyphenol content of cultivars in the monsoon season.

**Figure 6:**
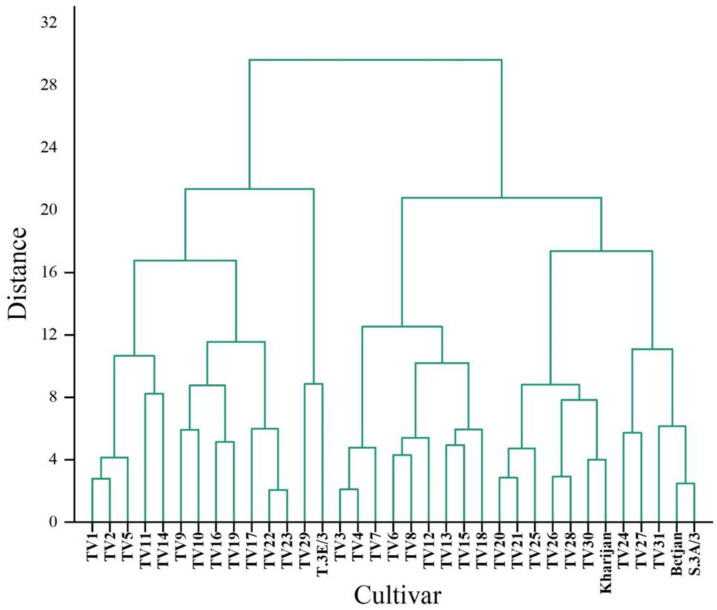
Dendrogram representing clustering of cultivars based on total polyphenol content of cultivars in autumn.

A hierarchical cluster analysis (HCA) was applied to identify the presence of homogenous groups among the cultivars based on TP content in each harvesting season separately (Figure 4, Figure 5 and Figure 6). HCA was applied to the distance-based idea that nearby cultivars are more related than those far away. For example, the dendrogram of TP content in the pre-monsoon season (Figure 4) presents different homogeneous groups such as TV2 and TV3, TV4 and TV5, etc.

The present study revealed that tea leaves contain the highest TP content in the monsoon season. Bhatia and Ullah also reported similar findings, where on average, TP content was found to be highest during the monsoon period, although an observation with individual cultivars may show differences in terms of high or low phenolic content [29]. Therefore, good-quality tea is expected to be produced during this period. The TP content decreases rapidly in the autumn, with a concomitant decrease in the tender shoots as growth retards. Polyphenols, more specifically catechins, also play a critical role in identifying the geographical origin, seasonal variation, and region-specific characteristics of processed tea [30,31,32]. The TP content of Sri Lankan tea was reported in the range between 153.08 and 287.88 mg g^−1^ [33], which supports the present findings.

In our previous study, it was observed that the five principal catechins contributed around 75% to the TP content of the cultivars [7]. Therefore, the variation in these catechins is primarily responsible for the observed variation in TP content with the harvesting season. These variations in TP content depend on the agro-climatic conditions of the tea plant such as sunlight, temperature, drainage, rainfall, and altitude [34,35,36,37,38]. The EGCG and ECG levels in cultivars were highest in the monsoon season, which could be related to increased temperature in the monsoon period [7]. Wen et al. observed enhanced levels of catechins in the tea shoots with an increase in exposure to sunlight and temperature [32].

### 3.2. Antioxidant Activity

The innumerable health benefits of tea have been attributed to its potent antioxidant activity. The antioxidant activity of tea leaves or green tea is primarily attributed to the flavonoids, more specifically, the catechin content [39,40,41,42]. Therefore, flavonoids can be termed as a biological antioxidant, which is defined as “any substance that when present at low concentrations compared to those of an oxidizable substrate, significantly delays or prevents oxidation of that substrate” [27]. Antioxidant flavonoids scavenge endogenously generated free radicals such as superoxide, peroxyl, and hydroxyl radicals. The ROS produced in the human body during biological combustion leads to oxidative stress by causing collective damage to proteins, lipids, and DNA [43]. Several reports have indicated that the polyphenolic compound present in tea prevents lipid peroxidation in phospholipid bilayers and biological systems and acts against tumorigenesis and DNA damage [44,45]. Oxidative degradation of lipids such as LDL oxidation by free metal ions in the arteries triggers the formation of atherosclerotic plaque, which leads to coronary heart diseases. Moreover, damaged proteins can undergo misfolding losing activity or possibly accumulation in the brain leading to neurodegenerative processes [46]. In Western countries, green tea consumption is driven by consumers’ growing awareness of well-being, which is closely related to its high antioxidant property [47]. Moreover, green tea is the second most preferred tea of consumers, accounting for around 15% of total world consumption [48]. The antioxidant activity of tea leaves or green tea can be attributed to catechins, rutin, hyperoside, quercetin 3-*O*-glucoside, kaempferol 3-*O*-glucoside, phenolic acids, and other organic acids [35,46].

A wide variety of methods have been frequently used to demonstrate the antioxidant activity of tea including DPPH, ABTS, FRAP (ferric-reducing antioxidant potential), and lipid peroxidation inhibition (LPI) assays. DPPH and ABTS assays are very convenient for measuring antioxidant activity due to the stability of their free radicals, simplicity, and the short time required for analysis. The degree of decoloration, caused by the reduction in the free radical by the phenolic compounds, which is directly proportional to the number of hydroxy groups present [49], is the measure of antioxidant activity. During the FRAP assay, a reduction of ferric to ferrous ion results in the formation of a colored ferrous-tripyridyltriazine complex. This color development is the measure of the antioxidant potential [27]. An LPI assay measures the ability of a tea bioactive to prevent the oxidative degradation of lipids. Rutin, a flavonoid, and caffeoyl derivatives (chlorogenic and caffeic acid) exhibit a DPPH free radical scavenging effect and inhibition of lipid peroxidation in a concentration-dependent way. Among these, chlorogenic acid was reported as the most potent antioxidant [50]. Methylxanthines, such as caffeine and its catabolic products xanthine and theobromine, can exhibit both antioxidant and pro-oxidant properties [40,50,51]. Amino acids such as L-theanine, L-histidine, L-alanine, L-tyrosine, L-isoleucine, L-leucine, and L-threonine have also been reported to affect the antioxidant activity of tea in either way [40,52]. Several amino acids form oligomers with relevant phenolic compounds, which could reduce the antioxidant activity [40]. Ma observed from a FRAP, a DPPH assay, and an ABTS assay of tea that antioxidant activity was significantly (*p* < 0.001) correlated with the contents of total flavonoids, polyphenols, free amino acids, soluble sugars, and catechins [52]. However, ellagic acid, quercetin, and kaempferol were reported to have a significant (*p* < 0.05) negative correlation to DPPH and ABTS activity, which is contrary to a few other reports [35,46]. Flavonoids (such as rutin, myricetin, taxifolin, and luteolin) and ellagic acid have also been reported as contributors to the FRAP activity of tea [51]. Catechins, free amino acids, 1,4,6-tri-*O*-galloyl-β-D-glucose, caffeine, and theobromine were observed to show significant (*p* < 0.01) positive correlations with the DPPH scavenging activity of tea [51].

#### 3.2.1. DPPH Scavenging Activity

The DPPH scavenging activity of tea cultivars varied per TP content. Table 2 depicts the antioxidant activity of cultivars in three seasons.

The DPPH activity was observed to be highest in the monsoon season owing to the higher level of polyphenolic contents. The cultivar TV1 exhibited the highest activity, with 187.10 mM TE per gram of sample, whereas the lowest activity was observed in the case of TV26 (168.34 mM TE g^−1^). This antioxidant property showed a significant positive correlation with the TP (R^2^ = 0.92, *p* ≤ 0.01), TC (R^2^ = 0.35, *p* ≤ 0.05), and ECG (R^2^ = 0.43, *p* ≤ 0.01) contents of cultivars (Figure 2). The DPPH activity of cultivars varied from 149.17 mM TE g^−1^ (TV8) to 171.25 mM TE g^−1^ (TV3) and from 143.98 mM TE g^−1^ (TV27) to 165.81 mM TE g^−1^ (TV9) in the pre-monsoon and autumn season, respectively. The correlation of the activity with TP contents was observed to be highly significant in both seasons (R^2^ = 0.90, *p* ≤ 0.01). Moreover, DPPH activity also had a significant correlation with the TC (pre-monsoon: R^2^ = 0.59; autumn: R^2^ = 0.64, *p* ≤ 0.01) and EGCG (pre-monsoon: R^2^ = 0.39; autumn: R^2^ = 0.43, *p* ≤ 0.05) contents (Figure 1 and Figure 3). Lv et al. reported DPPH scavenging activity in the range from 76.02 to 95.58% in different types of processed tea, which is very similar to the results of the current study (143.98–187.1 mM TE g^−1^, which is equivalent to 74.53–97.45%) [53]. Green tea processed from purple-leaf-colored tea cultivars of Kenya had DPPH activity in the range of 86.9% to 94.4% [54]. Other studies on the line also observed a significant positive correlation between DPPH scavenging activity with TP content [54,55]. In all three seasons, DPPH activity showed highly significant positive correlations with ABTS, FRAP, and LPI antioxidative assays with R^2^ values ranging from 0.73 to 0.96 (*p* ≤ 0.01) (Figure 1, Figure 2 and Figure 3). The clustering of cultivars for their DPPH scavenging activity was also performed using HCA in each harvesting season (Supplementary information, Figures S2–S4).

#### 3.2.2. ABTS Scavenging Activity

The ABTS activity of the cultivar TV3 was found to be highest in the pre-monsoon season, with 11,830.70 mM TE g^−1^. The activity of cultivar TV3 was significantly different (*p* ≤ 0.01) from all others except TV2 and TV29. The cultivar TV8 had the lowest activity, with 7609.17 mM TE g^−1^, which was significantly different from the others (*p* ≤ 0.01) (Table 3).

The ABTS activities showed a strong positive correlation with the TP (R^2^ = 0.94, *p* ≤ 0.01), TC (R^2^ = 0.56, *p* ≤ 0.01), galloylated catechin (R^2^ = 0.46, *p* ≤ 0.01), and EGCG (R^2^ = 0.36, *p* ≤ 0.05) contents of the cultivars in the pre-monsoon season (Figure 1). In the monsoon season, cultivar TV1 showed the highest ABTS scavenging activity (12,134.37 mM TE g^−1^). The activity of cultivar TV1 was significantly different (*p* ≤ 0.01) from all others except TV11, which had a similar level. The activities of the cultivars TV21, TV26, and TV27 were on the lower side, with values ranging from 9072.38 to 9090.30 mM TE g^−1^, which were significantly different (*p* ≤ 0.01) from the remaining cultivars (Table 3). The TP, TC, galloylated catechin, and ECG contents had a significant (*p* ≤ 0.01) positive correlation with ABTS activity, with R^2^ values of 0.96, 0.46, 0.50, and 0.49, respectively (Figure 2). In autumn, ABTS activity ranged from 5289.92 mM TE g^−1^ (TV27) to 10,086.75 mM TE g^−1^ (TV9) (Table 3). Both extreme activities were significantly different (*p* ≤ 0.01) from all other cultivars. The ABTS activities showed a strong positive correlation with the TP (R^2^ = 0.94, *p* ≤ 0.01), TC (R^2^ = 0.56, *p* ≤ 0.01), galloylated catechin (R^2^ = 0.66, *p* ≤ 0.01), and EGCG (R^2^ = 0.50, *p* ≤ 0.01) contents of the cultivars in autumn (Figure 3). In the current study, a significant linear correlation of ABTS activity with TP content was observed compared with that in previous studies conducted by Rusak et al. [56], with R^2^ = 0.90, and Carloni et al. [20], with R^2^ = 0.87. ABTS activity in all three seasons showed highly significant positive correlations with the DPPH, FRAP, and LPI assays, with R^2^ values ranging from 0.79 to 0.96 (*p* ≤ 0.01) (Figure 1, Figure 2 and Figure 3). The clustering of cultivars for their ABTS scavenging activity was also carried out using HCA in each harvesting season (Supplementary information, Figures S5–S7).

#### 3.2.3. Ferric-Reducing Antioxidant Potential

In the pre-monsoon season, the FRAP of green leaves varied from 6201.94 mM Fe^2+^ g^−1^ in TV7 to 8944.58 mM Fe^2+^ g^−1^ in TV3 (Table 4). The cultivars TV2 and TV29 had similar FRAP activities to that of TV3. The FRAP activities of the remaining cultivars were significantly different (*p* ≤ 0.01) from these cultivars. A highly significant (*p* ≤ 0.01) positive correlation of FRAP activities was observed with the TP (R^2^ = 0.87), TC (R^2^ = 0.69), galloylated catechin (R^2^ = 0.52), and EGCG (R^2^ = 0.48) contents of the cultivars (Figure 1). In addition, the non-galloylated catechin and EGC content also showed a significant (*p* ≤ 0.05) positive correlation with FRAP activity. The cultivars showed the highest potential for reducing ferric ions in the monsoon season. This higher activity is a manifestation of a higher level of phenolic content in the monsoon as compared with the other two seasons. The FRAP values were observed to express a highly significant (*p* ≤ 0.01) positive correlation with TP content (R^2^ = 0.92). Moreover, the galloylated catechin, TC, EGCG, and ECG contents also had a significant (*p* ≤ 0.05) positive correlation with FRAP activities, with R^2^ values ranging from 0.34 to 0.50 (Figure 2). In the monsoon season, the FRAP of cultivars varied from 4912.36 mM Fe^2+^ g^−1^ in TV27 to 8973.19 mM Fe^2+^ g^−1^ in TV1, expressing an 82% variation between the highest- and lowest-ranked cultivars (Table 4). The highest activity in TV1 was significantly different (*p* ≤ 0.01) from all other cultivars. On the other hand, TV21 and TV27 had comparable low levels of FRAP with TV26. In autumn, FRAP varied between 4186.37 mM Fe^2+^ g^−1^ in TV27 and 10,980.31 mM Fe^2+^ g^−1^ in TV9, which expressed a 162% variation between the two extremes (Table 4). Both these cultivars were significantly different (*p* ≤ 0.01) from the remaining cultivars. In autumn also, the FRAP values observed a highly significant (*p* ≤ 0.01) positive correlation with the TP (R^2^ = 0.92), galloylated catechin (R^2^ = 0.70), TC (R^2^ = 0.58), EGCG (R^2^ = 0.46) and ECG (R^2^ = 0.55) contents of cultivars (Figure 3). Moreover, the caffeine levels in cultivars also had a significant (*p* ≤ 0.05) positive correlation. Rusak et al. reported a significant positive correlation of FRAP activity with TP content (R^2^ = 0.89 and 0.94 for two types of tea), which supports the current findings [56]. The FRAP activities throughout the harvesting period of tea cultivars had a highly significant positive correlation (R^2^ = 0.78–0.93, *p* ≤ 0.01) with DPPH, ABTS, and LPI antioxidant measures (Figure 1, Figure 2 and Figure 3). The clustering of cultivars for their FRAP activity was also performed using HCA in each harvesting season (Supplementary information, Figures S8–S10).

#### 3.2.4. Lipid Peroxidation Inhibition Activity

In the pre-monsoon season, LPI activity ranged from 51.25% on TV9 to 78.92% on TV3 (Table 5). The cultivars TV2, TV29, and Betjan also exhibited similar levels of activity as that of TV3. The activity of this group of cultivars at the higher extreme was significantly different (*p* ≤ 0.01) from the remaining cultivars. The LPI activity demonstrated a highly significant (*p* ≤ 0.01) positive correlation with TP content (R^2^ = 0.83) and a significant (*p* ≤ 0.05) positive correlation with the TC, galloylated catechin, and EGCG contents, with R^2^ values varying from 0.34 to 0.42 (Figure 1). The LPI activity in the monsoon ranged from 60.24% in TV26 to 74.36% in TV1 (Table 5). These inhibitory activities had significant positive correlations with the TP (R^2^ = 0.84, *p* ≤ 0.01), ECG (R^2^ = 0.42, *p* ≤ 0.05), and galloylated catechin (R^2^ = 0.41, *p* ≤ 0.05) contents of cultivars (Figure 2). In autumn, cultivar TV9 exhibited the highest LPI activity at 71.39%, whereas TV26 exhibited the lowest activity at 56.85% (Table 5). LPI activities expressed a highly significant (*p* ≤ 0.01) positive correlation with the contents of TP (R^2^ = 0.88), galloylated catechin (R^2^ = 0.59), TC (R^2^ = 0.49), and ECG (R^2^ = 0.54). Moreover, the EGCG and caffeine levels had a significant (*p* ≤ 0.05) positive correlation with this antioxidant activity (Figure 3). The LPI activity throughout the harvesting seasons of the year observed highly significant (*p* ≤ 0.01) correlations with DPPH, ABTS, and FRAP activities (R^2^ = 0.73–0.85) (Figure 1, Figure 2 and Figure 3). The clustering of cultivars for their LPI activity was also performed using HCA in each harvesting season (Supplementary information, Figures S11–S13).

The antioxidant activity of tea is an outcome of a large group of biochemical compounds such as flavonoids, amino acids, purine alkaloids, phenolic acids, sugars, etc., of which the polyphenolic and catechin contents largely determine the activity. The synergistic effects of different compounds should be considered while demonstrating the antioxidant activity of tea.

## 4. Conclusions

In this study, the impact of harvesting season on the TP content and antioxidant activity of thirty-five tea cultivars of Northeast India has been reported. In the monsoon season, the TP content of cultivars reached the highest values as compared with the pre-monsoon and autumn. The cultivars TV1, TV11, TV23, and TV29 showed a higher content of TP. Weather conditions such as increased temperature, sufficient rainfall, sunlight, etc. possibly accelerate the biosynthesis of polyphenolic compounds, profoundly the catechins, leading to a comparatively higher level of TP in cultivars. The antioxidant activity expressed a significant (*p* ≤ 0.01) positive correlation with the TP content of the cultivars, with R^2^ ranging from 0.83 to 0.96. The higher level of TP content in the monsoon season was reflected in the higher antioxidant activity in that season. The current study demonstrates the health-beneficial potential of TTRI-developed tea cultivars considering their antioxidative properties. The data presented here may help in producing quality tea with a high polyphenolic content.

## Figures and Tables

**Table 1:** The total polyphenol content of tea cultivars in the pre-monsoon, monsoon, and autumn season.

Cultivar	Harvesting Season		
	Pre-Monsoon (mg g^−1^)	Monsoon (mg g^−1^)	Autumn (mg g^−1^)
TV1	234.10 ± 11.47 ^efghi^	283.53 ± 3.91 ^a^	207.37 ± 3.59 ^bcde^
TV2	254.07 ± 6.94 ^ab^	260.63 ± 6.29 ^cdefg^	204.77 ± 4.20 ^cdef^
TV3	256.77 ± 2.28 ^a^	261.83 ± 3.58 ^bcdefg^	192.73 ± 3.58 ^fghij^
TV4	250.77 ± 3.96 ^abc^	243.37 ± 2.34 ^ijklmn^	190.90 ± 2.82 ^ghijk^
TV5	247.07 ± 5.34 ^abcd^	256.37 ± 3.02 ^defghi^	207.80 ± 7.45 ^bcde^
TV6	225.73 ± 2.74 ^hijk^	248.60 ± 7.89 ^ghijklm^	179.23 ± 3.55 ^klm^
TV7	229.03 ± 5.27 ^ghi^	240.63 ± 0.99 ^lmn^	194.90 ± 4.92 ^efghi^
TV8	197.87 ± 6.19 ^m^	253.33 ± 3.86 ^efghijkl^	183.03 ± 2.98 ^hijklm^
TV9	221.60 ± 8.25 ^ijk^	242.17 ± 5.83 ^jklmn^	223.50 ± 4.39 ^a^
TV10	239.87 ± 6.65 ^cdefg^	249.87 ± 9.51 ^fghijklm^	217.67 ± 5.95 ^abc^
TV11	225.97 ± 3.46 ^hijk^	274.53 ± 6.94 ^ab^	209.67 ± 10.03 ^bcd^
TV12	244.73 ± 2.84 ^abcde^	252.40 ± 3.59 ^efghijkl^	180.70 ± 1.85 ^jklm^
TV13	230.07 ± 3.41 ^fghi^	258.43 ± 1.82 ^defgh^	186.93 ± 6.00 ^hijkl^
TV14	215.13 ± 3.25 ^jkl^	250.37 ± 7.03 ^fghijklm^	202.00 ± 3.67 ^defg^
TV15	236.73 ± 7.72 ^defgh^	254.47 ± 3.69 ^efghijk^	191.43 ± 2.36 ^ghijk^
TV16	234.97 ± 3.68 ^defgh^	264.30 ± 7.17 ^bcde^	219.00 ± 4.85 ^ab^
TV17	244.23 ± 1.34 ^abcde^	255.43 ± 2.87 ^defghij^	211.67 ± 6.12 ^abcd^
TV18	198.40 ± 4.78 ^m^	238.50 ± 8.93 ^mn^	185.47 ± 5.54 ^hijkl^
TV19	234.47 ± 2.57 ^defgh^	251.23 ± 2.15 ^efghijklm^	223.17 ± 8.31 ^a^
TV20	251.07 ± 0.95 ^abc^	255.77 ± 0.86 ^defghi^	196.10 ± 3.97 ^efgh^
TV21	203.73 ± 3.20 ^lm^	230.57 ± 4.72 ^n^	193.43 ± 2.77 ^fghij^
TV22	234.87 ± 4.37 ^defgh^	262.97 ± 5.84 ^bcdef^	213.00 ± 5.85 ^abcd^
TV23	241.10 ± 1.13 ^cdefg^	272.50 ± 3.23 ^abc^	214.80 ± 10.76 ^abcd^
TV24	208.50 ± 4.72 ^lm^	238.23 ± 3.32 ^mn^	174.83 ± 3.12 ^lm^
TV25	242.87 ± 4.04 ^bcde^	241.53 ± 2.50 ^klmn^	195.07 ± 3.21 ^efgh^
TV26	208.67 ± 2.18 ^lm^	238.50 ± 0.89 ^mn^	190.07 ± 7.44 ^ghijk^
TV27	226.83 ± 5.90 ^hij^	240.63 ± 3.93 ^lmn^	169.97 ± 1.82 ^m^
TV28	240.73 ± 2.01 ^cdefg^	245.63 ± 6.81 ^hijklm^	187.93 ± 1.90 ^hijkl^
TV29	254.63 ± 5.37 ^ab^	273.00 ± 5.56 ^abc^	206.93 ± 3.72 ^bcde^
TV30	221.77 ± 1.98 ^ijk^	246.20 ± 5.35 ^hijklm^	196.23 ± 7.21 ^efgh^
TV31	243.43 ± 2.57 ^bcde^	268.23 ± 4.43 ^bcd^	176.73 ± 3.19 ^lm^
Betjan	252.30 ± 0.42 ^abc^	256.13 ± 2.87 ^defghi^	183.13 ± 3.59 ^hijklm^
Kharijan	242.50 ± 1.88 ^bcdef^	263.27 ± 3.58 ^bcdef^	193.60 ± 7.78 ^fghij^
S.3E/3	214.10 ± 3.32 ^kl^	245.93 ± 3.02 ^hijklm^	181.70 ± 2.45 ^ijklm^
T.3E/3	243.67 ± 2.44 ^bcde^	250.30 ± 0.87 ^fghijklm^	213.43 ± 2.41 ^abcd^

Values are ‘mean ± SE’ of independent triplicate measurements. A same superscript symbol within a column denotes insignificant, whereas a different symbol denotes a significant difference. Means were compared using Tukey’s multiple comparison tests at *p* ≤ 0.05.

**Table 2:** DPPH activity of tea cultivars in the pre-monsoon, monsoon, and autumn seasons.

Cultivar	Harvesting Season		
	Pre-Monsoon (mM TE g^−1^)	Monsoon (mM TE g^−1^)	Autumn (mM TE g^−1^)
TV1	159.34 ± 1.60 ^fghijk^	187.10 ± 5.84 ^a^	159.66 ± 5.99 ^abcde^
TV2	170.38 ± 2.94 ^abc^	176.44 ± 6.35 ^cdefghi^	158.87 ± 5.74 ^abcde^
TV3	171.25 ± 2.21 ^a^	176.60 ± 6.48 ^cdefghi^	155.20 ± 3.36 ^abcdef^
TV4	169.48 ± 3.67 ^abcd^	172.60 ± 1.87 ^fghi^	160.98 ± 1.41 ^abcd^
TV5	169.25 ± 3.49 ^abcde^	175.81 ± 5.01 ^cdefghi^	152.74 ± 5.00 ^bcdef^
TV6	156.41 ± 4.66 ^hijkl^	172.94 ± 4.48 ^efghi^	150.47 ± 5.30 ^cdef^
TV7	159.00 ± 5.21 ^fghijk^	174.30 ± 2.48 ^defghi^	155.95 ± 3.85 ^abcde^
TV8	149.17 ± 4.44 ^l^	174.49 ± 0.29 ^defghi^	160.14 ± 5.59 ^abcde^
TV9	155.54 ± 3.81 ^ijkl^	171.73 ± 0.87 ^fghi^	165.81 ± 4.97 ^a^
TV10	161.54 ± 1.86 ^cdefghij^	173.57 ± 1.78 ^efghi^	164.98 ± 2.83 ^a^
TV11	168.59 ± 2.58 ^abcde^	186.12 ± 5.64 ^ab^	160.99 ± 4.80 ^abcd^
TV12	157.78 ± 3.02 ^ghijkl^	173.95 ± 1.68 ^defghi^	149.17 ± 0.73 ^ef^
TV13	159.06 ± 4.49 ^fghijk^	176.43 ± 2.39 ^cdefghi^	152.81 ± 2.80 ^bcdef^
TV14	155.87 ± 2.66 ^hijkl^	173.74 ± 1.84 ^efghi^	158.45 ± 5.64 ^abcde^
TV15	161.25 ± 2.78 ^defghij^	174.56 ± 0.57 ^defghi^	153.33 ± 1.91 ^bcdef^
TV16	161.04 ± 2.40 ^defghij^	179.58 ± 2.85 ^abcdef^	165.00 ± 2.53 ^a^
TV17	167.69 ± 0.83 ^abcdef^	174.86 ± 0.66 ^defghi^	161.67 ± 2.74 ^abc^
TV18	151.03 ± 4.73 ^kl^	170.64 ± 3.51 ^ghi^	151.85 ± 4.97 ^cdef^
TV19	159.64 ± 2.31 ^fghijk^	173.53 ± 1.33 ^efghi^	165.22 ± 4.65 ^a^
TV20	169.73 ± 3.16 ^abcd^	174.88 ± 3.82 ^defghi^	156.24 ± 6.05 ^abcde^
TV21	153.42 ± 5.23 ^jkl^	169.80 ± 5.29 ^hi^	155.39 ± 2.56 ^abcde^
TV22	160.52 ± 4.18 ^efghij^	178.16 ± 2.71 ^bcdefgh^	163.93 ± 2.27 ^ab^
TV23	162.63 ± 2.46 ^abcdefghi^	182.73 ± 4.00 ^abcd^	163.99 ± 2.34 ^ab^
TV24	153.90 ± 4.04 ^ijkl^	170.07 ± 3.54 ^ghi^	149.81 ± 3.20 ^def^
TV25	166.01 ± 2.07 ^abcdefg^	171.27 ± 3.62 ^fghi^	156.80 ± 1.88 ^abcde^
TV26	153.92 ± 4.97 ^ijkl^	168.34 ± 1.82 ^i^	156.32 ± 2.77 ^abcde^
TV27	153.90 ± 4.04 ^ijkl^	168.84 ± 3.93 ^i^	143.98 ± 5.85 ^f^
TV28	162.24 ± 3.38 ^bcdefghij^	172.76 ± 0.29 ^fghi^	152.27 ± 2.81 ^cdef^
TV29	170.45 ± 3.85 ^ab^	184.53 ± 4.10 ^abc^	159.37 ± 5.65 ^abcde^
TV30	156.14 ± 5.64 ^hijkl^	172.79 ± 3.35 ^fghi^	156.71 ± 0.50 ^abcde^
TV31	166.88 ± 1.73 ^abcdef^	175.54 ± 2.66 ^defghi^	149.88 ± 1.80 ^def^
Betjan	169.76 ± 3.38 ^abcd^	181.74 ± 1.92 ^abcde^	151.09 ± 6.35 ^cdef^
Kharijan	164.45 ± 1.40 ^abcdefgh^	178.74 ± 3.90 ^abcdefg^	157.42 ± 4.48 ^abcde^
S.3E/3	154.34 ± 3.06 ^ijkl^	171.56 ± 3.24 ^fghi^	150.17 ± 3.93 ^def^
T.3E/3	167.24 ± 3.59 ^abcdef^	173.58 ± 3.36 ^efghi^	163.97 ± 3.08 ^ab^

Values are ‘mean ± SE’ of independent triplicate measurements. A same superscript symbol within a column denotes an insignificant difference, whereas a different symbol denotes a significant difference. Means were compared using Tukey’s multiple comparison tests at *p* ≤ 0.05.

**Table 3:** ABTS activity of tea cultivars in the pre-monsoon, monsoon, and autumn seasons.

Cultivar	Harvesting Season		
	Pre-Monsoon (mM TE g^−1^)	Monsoon (mM TE g^−1^)	Autumn (mM TE g^−1^)
TV1	10,213.90 ± 44.59 ^jk^	12,134.37 ± 59.27 ^a^	8536.72 ± 42.97 ^gh^
TV2	11,750.73 ± 122.38 ^a^	11,209.64 ± 65.66 ^e^	8469.52 ± 42.34 ^hi^
TV3	11,830.70 ± 23.44 ^a^	11,332.75 ± 64.68 ^d^	7941.42 ± 38.99 ^k^
TV4	11,199.32 ± 63.59 ^bc^	9987.55 ± 40.30 ^opq^	7734.20 ± 32.81 ^l^
TV5	11,090.58 ± 40.89 ^c^	11,105.18 ± 26.78 ^f^	8556.97 ± 54.25 ^gh^
TV6	9438.97 ± 42.61 ^mn^	10,244.80 ± 41.36 ^klm^	6122.44 ± 42.43 ^r^
TV7	9896.91 ± 78.22 ^l^	9623.12 ± 67.65 ^t^	8259.00 ± 57.17 ^j^
TV8	7609.17 ± 30.97 ^u^	10,834.63 ± 49.98 ^i^	7069.59 ± 56.08 ^n^
TV9	9284.97 ± 23.36 ^nopq^	9844.45 ± 50.79 ^rs^	10,086.75 ± 75.96 ^a^
TV10	10,535.64 ± 49.48 ^hi^	10,288.03 ± 31.31 ^kl^	8569.55 ± 54.51 ^gh^
TV11	11,036.23 ± 73.57 ^cd^	12,106.78 ± 40.57 ^a^	9409.87 ± 28.12 ^c^
TV12	9554.68 ± 38.52 ^m^	10,894.74 ± 37.60 ^hi^	6907.59 ± 74.88
TV13	10,177.82 ± 41.89 ^k^	11,107.99 ± 31.66 ^f^	7391.38 ± 39.41 ^m^
TV14	9317.90 ± 42.28 ^nopq^	10,495.22 ± 17.51 ^j^	8319.40 ± 45.88 ^j^
TV15	10,384.67 ± 98.09 ^ij^	10,835.21 ± 20.27 ^i^	7727.59 ± 67.11 ^l^
TV16	10,337.57 ± 125.60 ^jk^	11,546.60 ± 63.66 ^c^	9428.12 ± 17.45 ^c^
TV17	10,884.62 ± 105.81 ^de^	10,961.05 ± 25.56 ^gh^	8617.78 ± 55.55 ^g^
TV18	7981.63 ± 18.99 ^t^	9608.17 ± 35.02 ^t^	7510.05 ± 17.18 ^m^
TV19	10,251.11 ± 44.16 ^jk^	10,502.50 ± 46.99 ^j^	9794.48 ± 68.13 ^b^
TV20	11,284.62 ± 42.09 ^b^	11,022.12 ± 44.63 ^fg^	8315.00 ± 29.81 ^j^
TV21	8660.34 ± 36.68 ^s^	9090.30 ± 26.44 ^u^	7941.83 ± 49.15 ^k^
TV22	10,263.16 ± 26.90 ^jk^	11,462.58 ± 73.97 ^c^	8757.48 ± 60.83 ^f^
TV23	10,548.34 ± 43.01 ^ghi^	11,856.82 ± 54.73 ^b^	9256.61 ± 31.01 ^d^
TV24	8960.63 ± 98.69 ^r^	10,065.63 ± 41.88 ^nop^	5870.09 ± 35.89 ^s^
TV25	10,724.55 ± 20.53 ^efg^	9773.85 ± 25.68 ^s^	8286.62 ± 28.79 ^j^
TV26	9136.25 ± 23.84 ^qr^	9072.38 ± 38.60 ^u^	8309.97 ± 43.06 ^j^
TV27	9592.67 ± 103.35 ^m^	9078.26 ± 62.81 ^u^	5289.92 ± 69.13 ^t^
TV28	10,541.79 ± 26.68 ^hi^	10,155.32 ± 31.57 ^mn^	7488.44 ± 31.11 ^m^
TV29	11,779.27 ± 131.91 ^a^	11,900.80 ± 61.51 ^b^	8593.12 ± 23.99 ^gh^
TV30	9415.77 ± 51.71 ^mnop^	10,221.55 ± 19.84 ^lm^	8395.70 ± 30.19 ^ij^
TV31	10,761.24 ± 33.88 ^ef^	11,853.46 ± 38.07 ^b^	5887.38 ± 28.84 ^s^
Betjan	11,376.72 ± 84.12 ^b^	11,073.47 ± 43.48 ^f^	7200.65 ± 39.48 ^n^
Kharijan	10,688.07 ± 37.02 ^fgh^	11,505.97 ± 66.72 ^c^	7950.53 ± 52.96 ^k^
S.3E/3	9239.10 ± 41.76 ^opq^	9911.98 ± 51.28 ^qr^	6909.38 ± 52.37 ^op^
T.3E/3	10,830.18 ± 18.42 ^ef^	10,330.13 ± 47.61 ^k^	8918.40 ± 72.22 ^e^

Values are ‘mean ± SE’ of independent triplicate measurements. A same superscript symbol within a column denotes an insignificant difference, whereas a different symbol denotes a significant difference. Means were compared using Tukey’s multiple comparison tests at *p* ≤ 0.05.

**Table 4:** FRAP activity of tea cultivars in the pre-monsoon, monsoon, and autumn seasons.

Cultivar	Harvesting Season		
	Pre-Monsoon (mM Fe^2+^ g^−1^)	Monsoon (mM Fe^2+^ g^−1^)	Autumn (mM Fe^2+^ g^−1^)
TV1	7680.99 ± 64.58 ^hi^	8973.19 ± 24.68 ^a^	7755.81 ± 38.60 ^gh^
TV2	8891.06 ± 22.66 ^a^	7595.40 ± 37.68 ^fg^	7486.92 ± 53.45 ^i^
TV3	8944.58 ± 33.70 ^a^	7702.81 ± 37.04 ^ef^	6515.63 ± 17.30 ^m^
TV4	8672.55 ± 39.61 ^c^	6393.48 ± 29.24 ^op^	7755.47 ± 35.10 ^gh^
TV5	8631.22 ± 27.95 ^c^	7553.77 ± 63.26 ^gh^	6354.74 ± 46.84 ^n^
TV6	7618.35 ± 20.46 ^ij^	6570.98 ± 36.58 ^mn^	5332.11 ± 22.67 ^s^
TV7	6201.94 ± 34.25 ^r^	5828.10 ± 48.42 ^t^	6857.73 ± 63.72 ^l^
TV8	7667.21 ± 51.40 ^hi^	6881.66 ± 108.68 ^k^	5562.48 ± 32.02 ^r^
TV9	7470.37 ± 30.47 ^k^	6248.21 ± 49.39	10,980.31 ± 73.43 ^a^
TV10	8155.93 ± 38.51 ^f^	6575.21 ± 38.72 ^mn^	8317.04 ± 59.09 ^d^
TV11	7637.45 ± 28.70 ^hi^	8664.02 ± 40.17 ^b^	7817.13 ± 77.37 ^gh^
TV12	8465.30 ± 35.00 ^d^	6765.89 ± 73.70 ^kl^	5363.22 ± 28.62 ^s^
TV13	7684.51 ± 37.84 ^hi^	7574.02 ± 46.50 ^g^	5830.80 ± 43.98 ^q^
TV14	7503.57 ± 52.58 ^k^	6620.53 ± 36.72 ^mn^	7331.04 ± 28.19 ^j^
TV15	8111.55 ± 47.26 ^f^	7022.65 ± 23.36 ^j^	6439.91 ± 52.38 ^mn^
TV16	7954.69 ± 34.21 ^g^	8071.08 ± 42.87 ^d^	8483.35 ± 44.01 ^c^
TV17	8429.11 ± 51.19 ^de^	5664.64 ± 25.40 ^u^	7831.67 ± 42.46 ^fgh^
TV18	6465.32 ± 36.59 ^q^	7026.72 ± 52.12 ^j^	5853.23 ± 49.62 ^q^
TV19	7738.12 ± 43.94 ^h^	6665.75 ± 41.21 ^lm^	10,320.67 ± 55.72 ^b^
TV20	8694.09 ± 55.52 ^bc^	7383.28 ± 48.52 ^i^	7184.65 ± 34.30 ^k^
TV21	6722.59 ± 32.77 ^op^	5171.70 ± 58.94 ^v^	6851.78 ± 32.46 ^l^
TV22	7921.48 ± 20.21 ^g^	7798.84 ± 34.05 ^e^	7873.81 ± 36.48 ^fg^
TV23	8340.76 ± 36.47 ^e^	8599.66 ± 47.44 ^b^	8065.90 ± 36.28 ^e^
TV24	6992.15 ± 64.82 ^n^	5570.90 ± 39.01 ^u^	4469.93 ± 31.96 ^u^
TV25	8396.85 ± 50.66 ^de^	6006.83 ± 41.88 ^s^	6895.30 ± 31.00 ^l^
TV26	7105.06 ± 40.67 ^m^	5144.33 ± 73.06 ^v^	7317.76 ± 29.41 ^j^
TV27	7659.95 ± 45.21 ^hi^	4912.36 ± 67.27 ^w^	4186.37 ± 49.43 ^v^
TV28	8196.51 ± 39.74 ^f^	6528.32 ± 47.05 ^n^	6035.48 ± 58.81 ^op^
TV29	8905.13 ± 35.05 ^a^	8662.66 ± 47.64 ^b^	7734.42 ± 25.68 ^h^
TV30	7530.76 ± 29.68 ^jk^	6552.48 ± 24.36 ^mn^	7270.60 ± 36.92 ^jk^
TV31	8407.24 ± 28.51 ^de^	8461.93 ± 26.23 ^c^	4826.03 ± 21.22 ^t^
Betjan	8775.82 ± 29.23 ^b^	7444.78 ± 57.69 ^hi^	5622.94 ± 42.51 ^r^
Kharijan	8391.82 ± 34.36 ^de^	7975.76 ± 53.67 ^d^	6831.83 ± 80.57 ^l^
S.3E/3	7213.09 ± 39.39 ^l^	6343.98 ± 30.03	5589.04 ± 22.73 ^r^
T.3E/3	8421.88 ± 43.49 ^de^	6582.97 ± 19.07 ^mn^	7946.12 ± 42.11 ^ef^

Values are ‘mean ± SE’ of independent triplicate measurements. A same superscript symbol within a column denotes an insignificant difference, whereas a different symbol denotes a significant difference. Means were compared using Tukey’s multiple comparison tests at *p* ≤ 0.05.

**Table 5:** LPI activity of tea cultivars in the pre-monsoon, monsoon, and autumn seasons.

Cultivar	Harvesting Season		
	Pre-Monsoon (%)	Monsoon (%)	Autumn (%)
TV1	64.16 ± 1.11 ^fghij^	74.36 ± 2.19 ^a^	65.27 ± 0.48 ^bcdef^
TV2	77.00 ± 0.34 ^a^	69.74 ± 3.57 ^bcdefg^	64.72 ± 1.33 ^bcdefgh^
TV3	78.92 ± 0.63 ^a^	70.88 ± 1.48 ^abcdef^	62.75 ± 1.54 ^defghijk^
TV4	71.52 ± 1.67 ^bc^	65.49 ± 1.94 ^hijklmn^	61.74 ± 2.47 ^fghijkl^
TV5	56.49 ± 0.61 ^lm^	68.89 ± 2.40 ^defghi^	65.18 ± 1.24 ^bedef^
TV6	61.63 ± 1.15 ^ijk^	66.06 ± 2.24 ^ghijklm^	58.39 ± 1.66 ^lm^
TV7	63.48 ± 1.00 ^ghij^	64.06 ± 1.94 ^klmnopq^	63.16 ± 1.83 ^defghijk^
TV8	58.65 ± 0.58 ^kl^	67.90 ± 1.74 ^fghijk^	59.21 ± 1.93 ^klm^
TV9	51.25 ± 4.62 ^n^	64.49 ± 2.10 ^jklmnop^	71.39 ± 1.18 ^a^
TV10	65.10 ± 1.52 ^efghi^	73.84 ± 2.39 ^a^	66.64 ± 1.58 ^bcd^
TV11	61.95 ± 1.53 ^hijk^	66.42 ± 1.56 ^ghijklm^	65.16 ± 1.16 ^bcdef^
TV12	70.37 ± 1.04 ^bcd^	67.94 ± 4.20 ^fghijk^	59.16 ± 1.24 ^klm^
TV13	63.95 ± 1.78 ^fghij^	69.09 ± 3.27 ^cdefgh^	60.74 ± 0.78 ^hijklm^
TV14	63.75 ± 0.95 ^fghij^	67.03 ± 2.27 ^fghijkl^	64.41 ± 1.12 ^bcdefgh^
TV15	64.71 ± 1.42 ^efhij^	68.01 ± 3.12 ^fghijk^	62.46 ± 2.09 ^efghijkl^
TV16	64.57 ± 0.85 ^fhij^	72.92 ± 2.44 ^abc^	67.94 ± 0.78 ^abc^
TV17	69.29 ± 2.19 ^bcd^	68.02 ± 1.18 ^fghijk^	65.19 ± 0.62 ^bcdef^
TV18	54.55 ± 1.61 ^mn^	63.82 ± 0.91 ^lmnopq^	60.07 ± 1.92 ^ijklm^
TV19	64.23 ± 1.64 ^fghij^	67.25 ± 2.04 ^fghijkl^	68.53 ± 0.71 ^ab^
TV20	72.30 ± 0.65 ^b^	68.61 ± 1.79 ^efghi^	63.60 ± 2.36 ^defghij^
TV21	55.66 ± 1.26 ^lm^	61.76 ± 2.71 ^nopq^	63.03 ± 2.75 ^defghijk^
TV22	64.23 ± 1.34 ^fghij^	70.92 ± 3.51 ^abcdef^	65.90 ± 0.86 ^bcde^
TV23	65.31 ± 1.91 ^efghi^	72.79 ± 1.80 ^abcd^	66.50 ± 1.03 ^bcde^
TV24	55.74 ± 0.98 ^lm^	62.97 ± 3.02 ^mnopq^	57.08 ± 1.35 ^m^
TV25	66.88 ± 1.62 ^defg^	64.95 ± 2.03 ^ijklmnop^	63.37 ± 0.40 ^defghij^
TV26	56.49 ± 0.61 ^lm^	60.24 ± 2.84 ^q^	56.85 ± 1.02 ^m^
TV27	63.43 ± 2.58 ^ghij^	61.44 ± 1.24 ^opq^	64.06 ± 0.82 ^cdefghi^
TV28	65.12 ± 2.22 ^efghi^	65.91 ± 2.03 ^ghijklm^	60.98 ± 3.05 ^ghijklm^
TV29	77.40 ± 0.48 ^a^	73.60 ± 2.06 ^ab^	64.90 ± 1.19 ^bcdefg^
TV30	61.04 ± 0.98 ^jk^	68.26 ± 2.87 ^fghij^	64.00 ± 2.48 ^cdefghi^
TV31	67.32 ± 1.57 ^def^	72.57 ± 1.17 ^abcde^	57.30 ± 1.64 ^m^
Betjan	76.73 ± 1.00 ^a^	68.64 ± 2.49 ^efghi^	59.59 ± 0.59 ^jklm^
Kharijan	65.48 ± 0.85 ^efgh^	70.92 ± 2.32 ^abcdef^	63.18 ± 0.72 ^defghijk^
S.3E/3	56.31 ± 1.61 ^lm^	65.38 ± 2.77 ^hijklmnop^	59.17 ± 0.37 ^klm^
T.3E/3	68.38 ± 2.04 ^cde^	66.83 ± 2.31 ^ghijklm^	65.73 ± 2.47 ^bcdef^

Values are ‘mean ± SE’ of independent triplicate measurements. A same superscript symbol within a column denotes an insignificant difference, whereas a different symbol denotes a significant difference. Means were compared using Tukey’s multiple comparison tests at *p* ≤ 0.05.

## Data Availability

Data is contained within the article or Supplementary Material.

## Supplementary Materials

The following supporting information can be downloaded at https://www.mdpi.com/article/10.3390/foods12173196/s1, Figure S1: Mean meteorological data of Borbheta Experimental Tea Estate during 2017; Figure S2: Dendrogram representing clustering of cultivars based on DPPH activity of cultivars in the pre-monsoon season; Figure S3: Dendrogram representing clustering of cultivars based on DPPH activity of cultivars in the monsoon season; Figure S4: Dendrogram representing clustering of cultivars based on DPPH activity of cultivars in autumn; Figure S5: Dendrogram representing clustering of cultivars based on ABTS activity of cultivars in the pre-monsoon season; Figure S6: Dendrogram representing clustering of cultivars based on ABTS activity of cultivars in the monsoon season; Figure S7: Dendrogram representing clustering of cultivars based on ABTS activity of cultivars in autumn; Figure S8: Dendrogram representing clustering of cultivars based on ferric-reducing antioxidant potential (FRAP) of cultivars in the pre-monsoon season; Figure S9: Dendrogram representing clustering of cultivars based on ferric-reducing antioxidant potential (FRAP) of cultivars in the monsoon season; Figure S10: Dendrogram representing clustering of cultivars based on ferric-reducing antioxidant potential (FRAP) of cultivars in autumn; Figure S11: Dendrogram representing clustering of cultivars based on lipid peroxidation inhibition activity of cultivars in the pre-monsoon season; Figure S12: Dendrogram representing clustering of cultivars based on lipid peroxidation inhibition activity of cultivars in the monsoon season; Figure S13: Dendrogram representing clustering of cultivars based on lipid peroxidation inhibition activity of cultivars in autumn.

## Abbreviations

ABTS: 2:2′-azinobis (3-ethylbenzothiazoline-6-sulfonic acid) diammonium salt; ANOVA: analysis of variance; +C: (+) catechin; DPPH: 2,2-diphenyl-1-picrylhydrazyl; EC: (-) epicatechin; ECG: (-) epicatechin-3-gallate; EGC: (-) epigallocatechin; EGCG: (-) epigallocatechin-3-gallate; FCP: Folin & Ciocalteu’s phenol, FRAP: ferric-reducing antioxidant potential; GC: galloylated catechin; LPI: lipid peroxidation inhibition; NGC: non-galloylated catechin; ROS: reactive oxygen species; SE: standard error; TC: total catechin; TE: trolox equivalent; TP: total polyphenol; TTRI: Tocklai Tea Research Institute; TV: Tocklai vegetative.
