# Supplementary material for: Impact of the Season on Total Polyphenol and Antioxidant Properties of Tea Cultivars of Industrial Importance in Northeast India
Source: Foods. 2023 Aug 24;12(17):3196. doi: 10.3390/foods12173196 (PMC10486918; doi:10.3390/foods12173196)
Supplement: Supplementary file 1 [file foods-12-03196-s001.zip › foods-2509714-supplementary.pdf]

Supplementary information

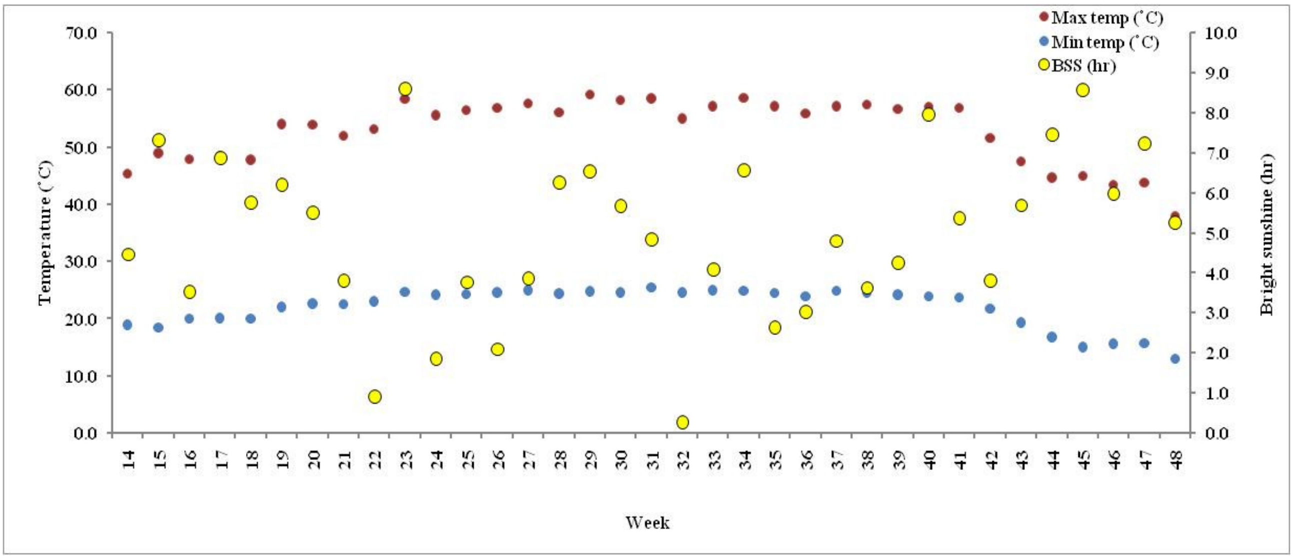

Figure S1. Mean meteorological data of Borbhetta Experimental Tea Estate during 2017.

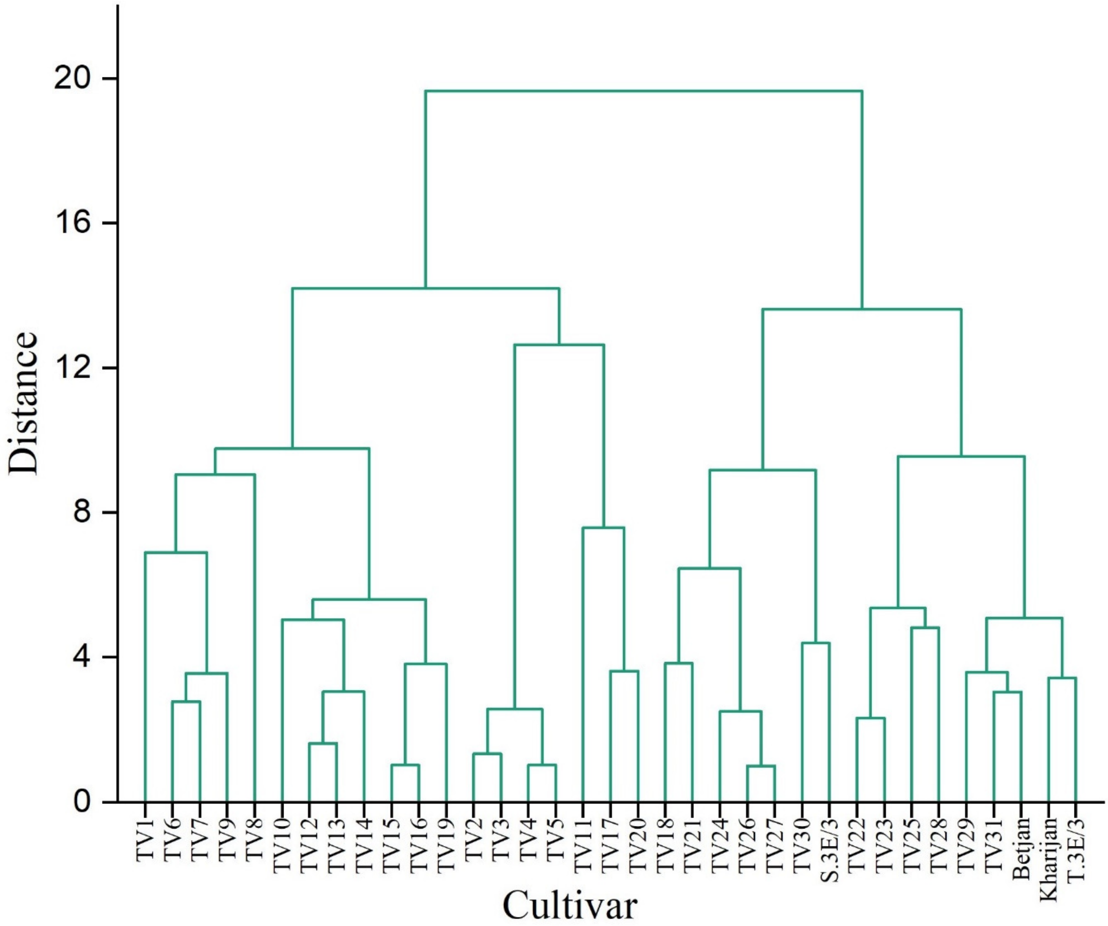

Figure S2. Dendrogram representing clustering of cultivars based on DPPH activity of cultivars in pre-monsoon.

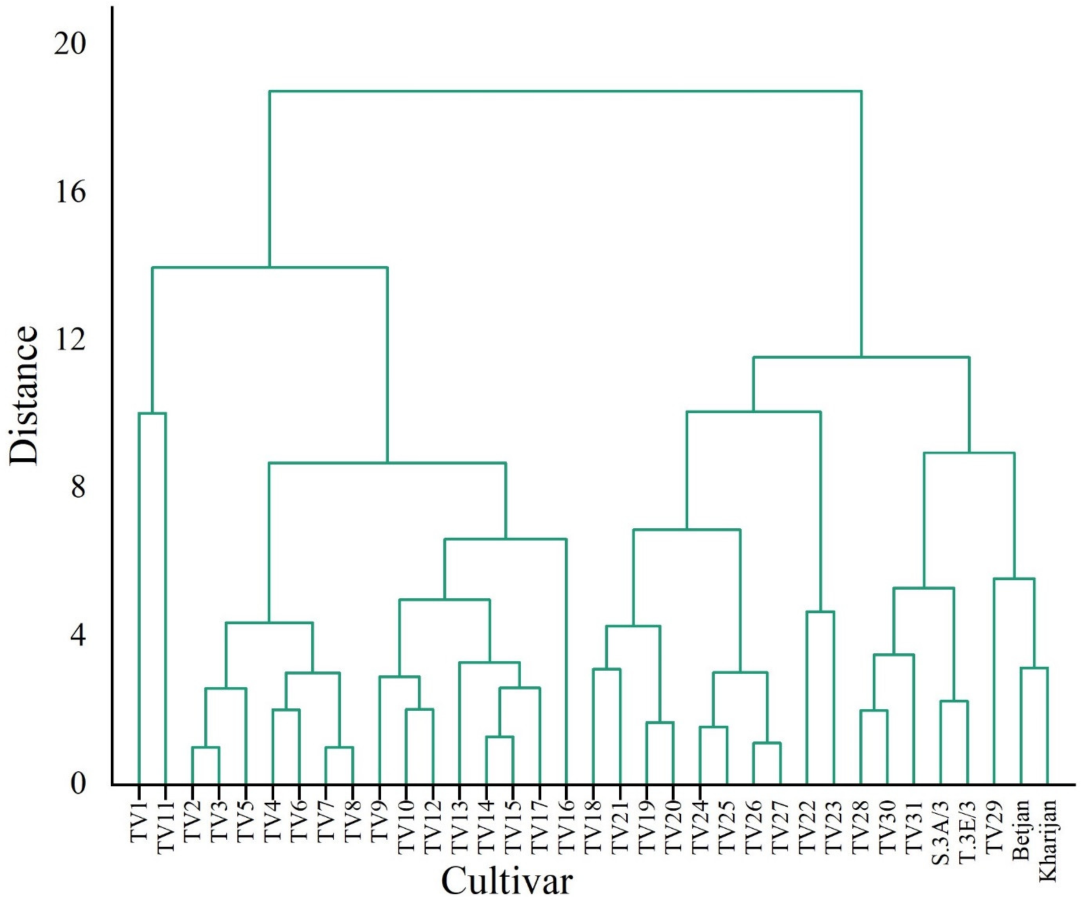

Figure S3. Dendrogram representing clustering of cultivars based on DPPH activity of cultivars in monsoon.

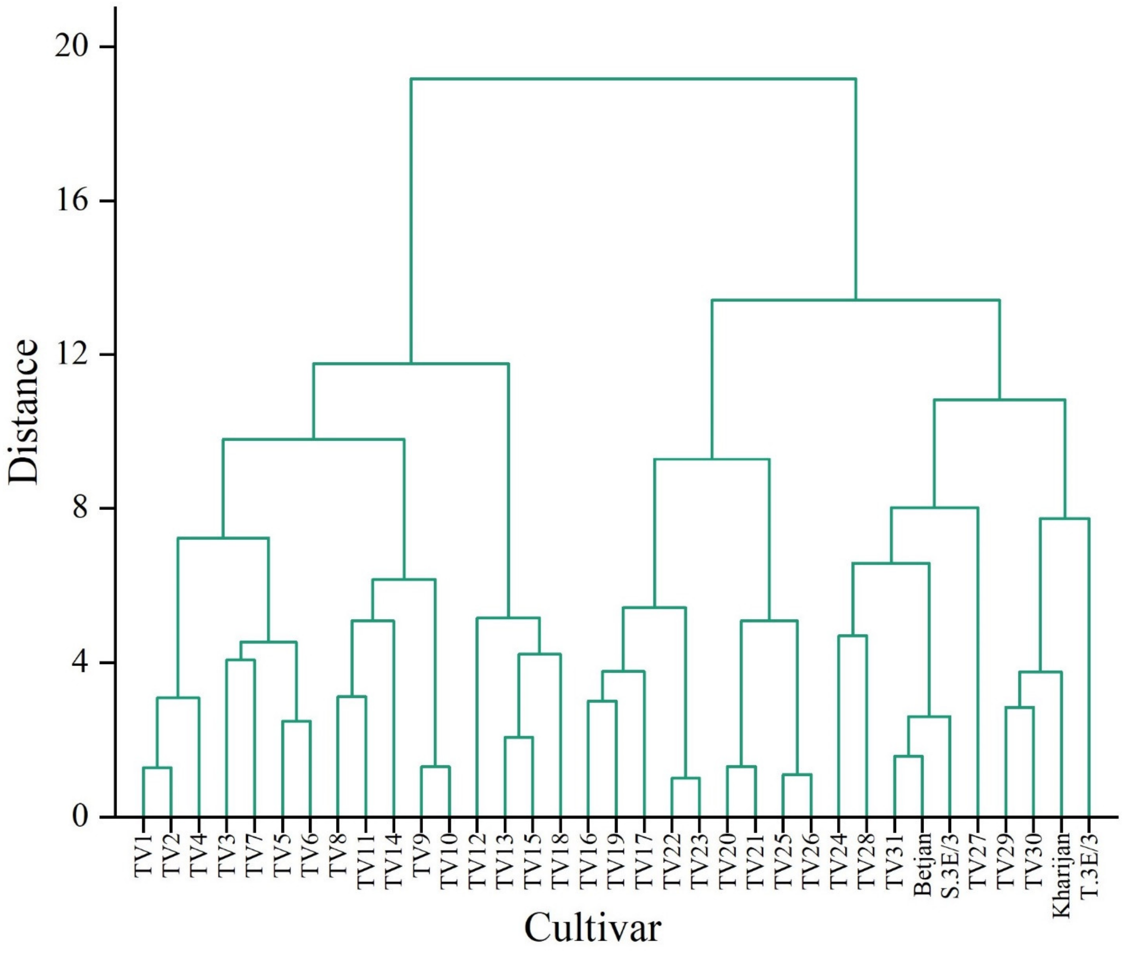

Figure S4. Dendrogram representing clustering of cultivars based on DPPH activity of cultivars in autumn.

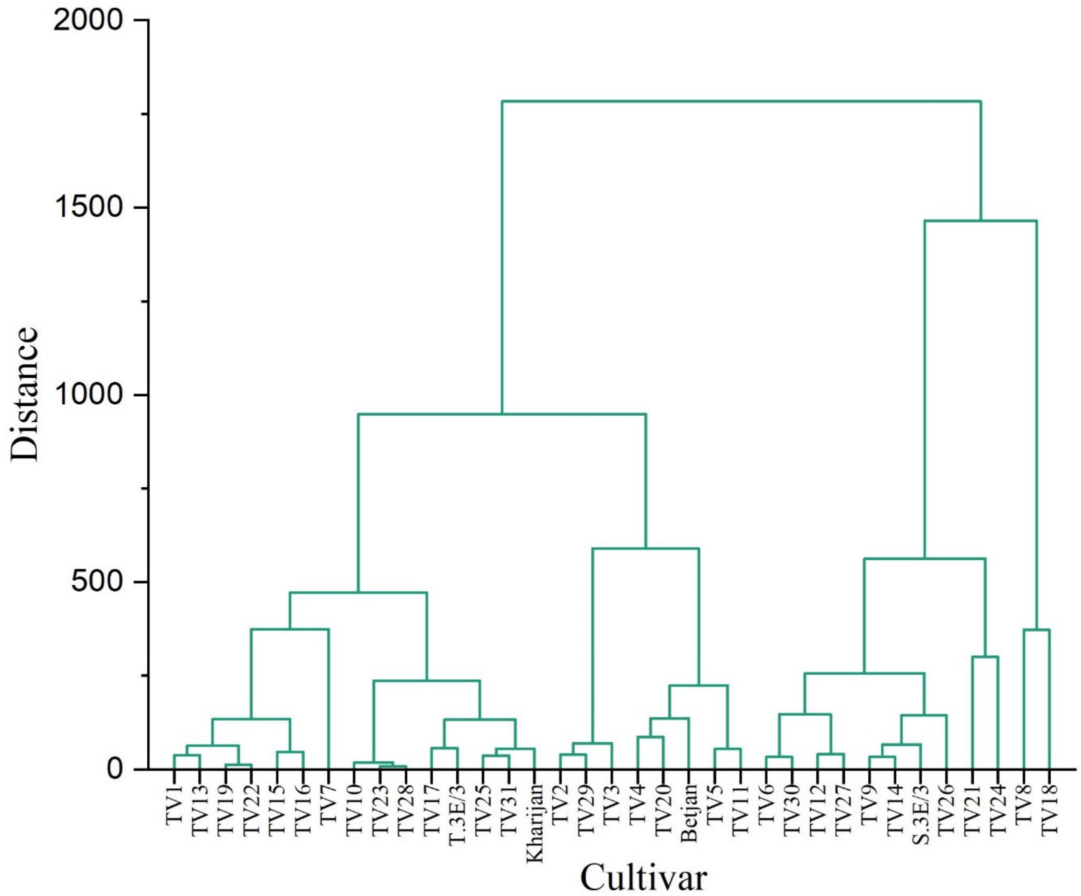

Figure S5. Dendrogram representing clustering of cultivars based on ABTS activity of cultivars in pre-monsoon.

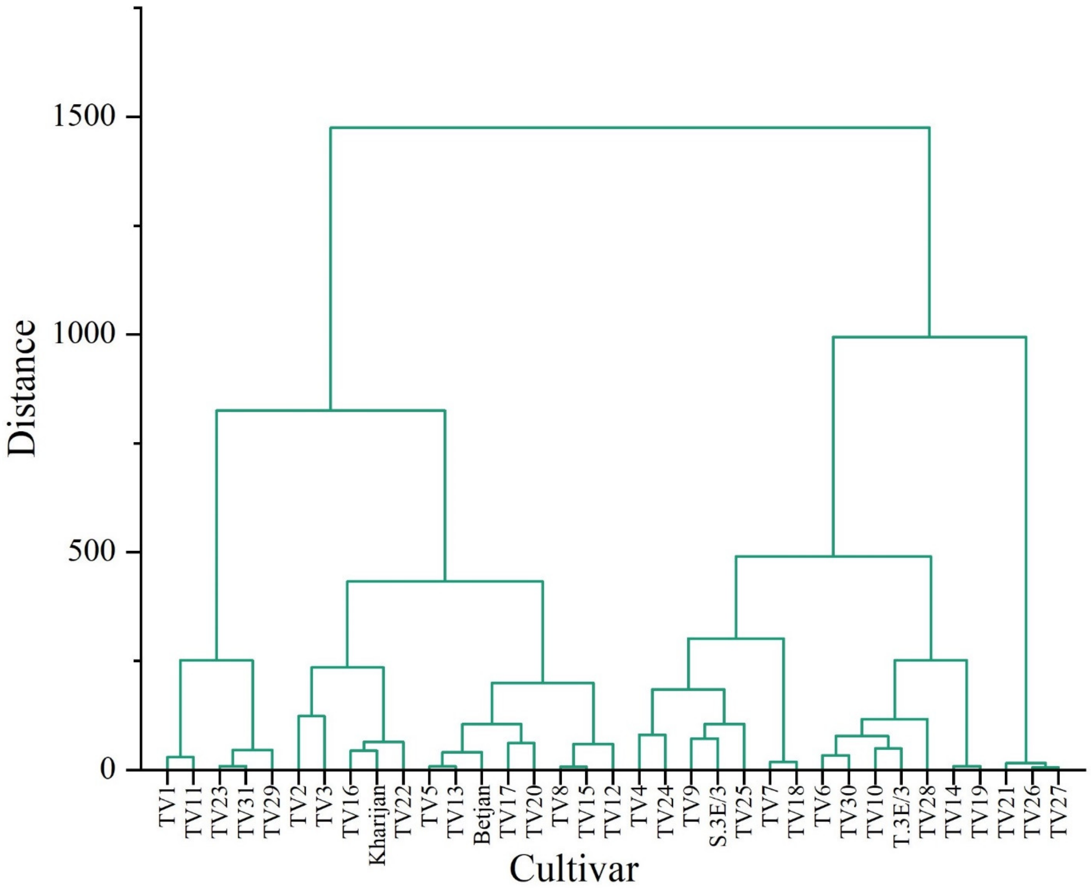

Figure S6. Dendrogram representing clustering of cultivars based on ABTS activity of cultivars in monsoon.

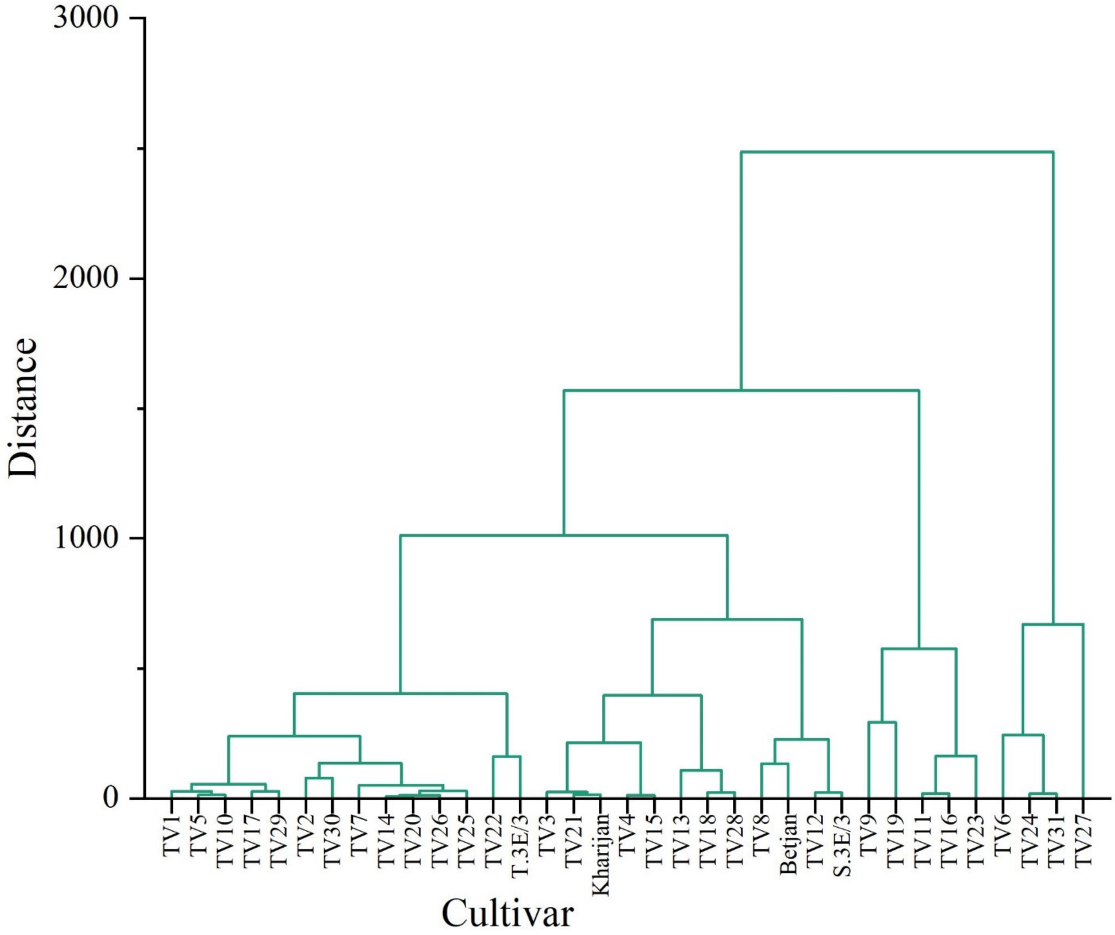

Figure S7. Dendrogram representing clustering of cultivars based on ABTS activity of cultivars in autumn.

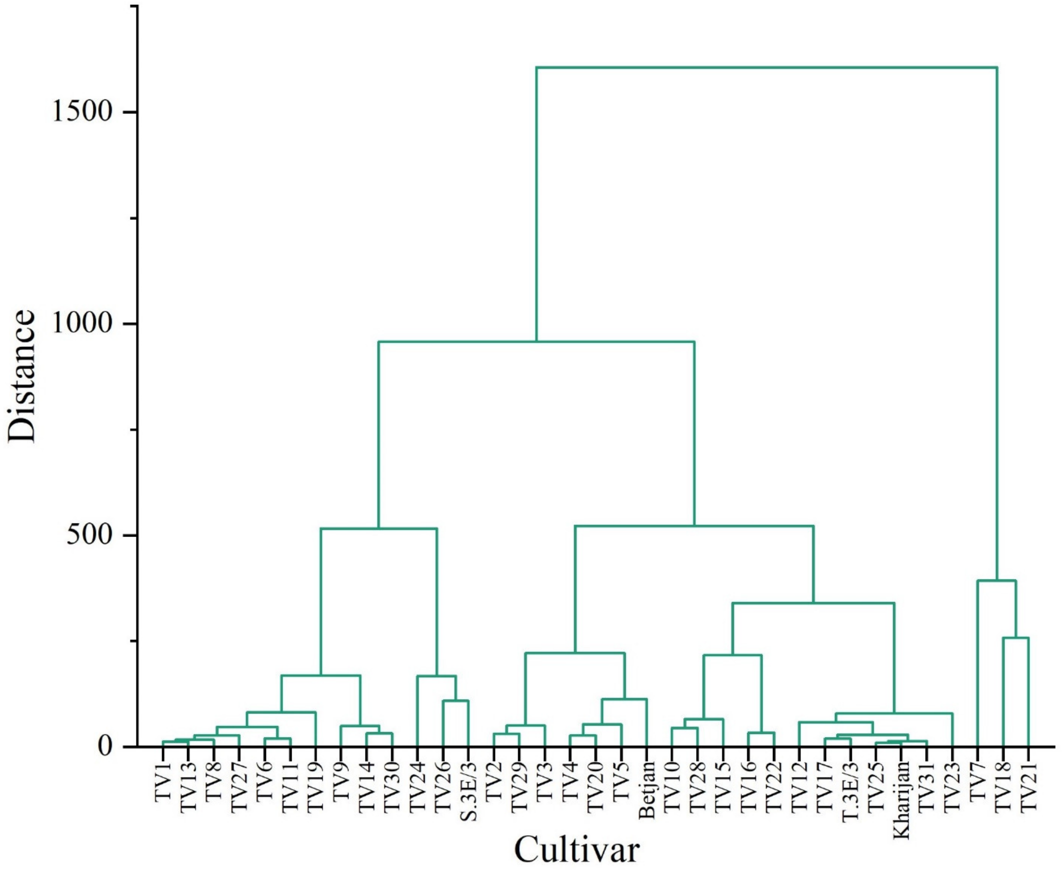

Figure S8. Dendrogram representing clustering of cultivars based on ferric reducing antioxidant potential (FRAP) of cultivars in pre-monsoon.

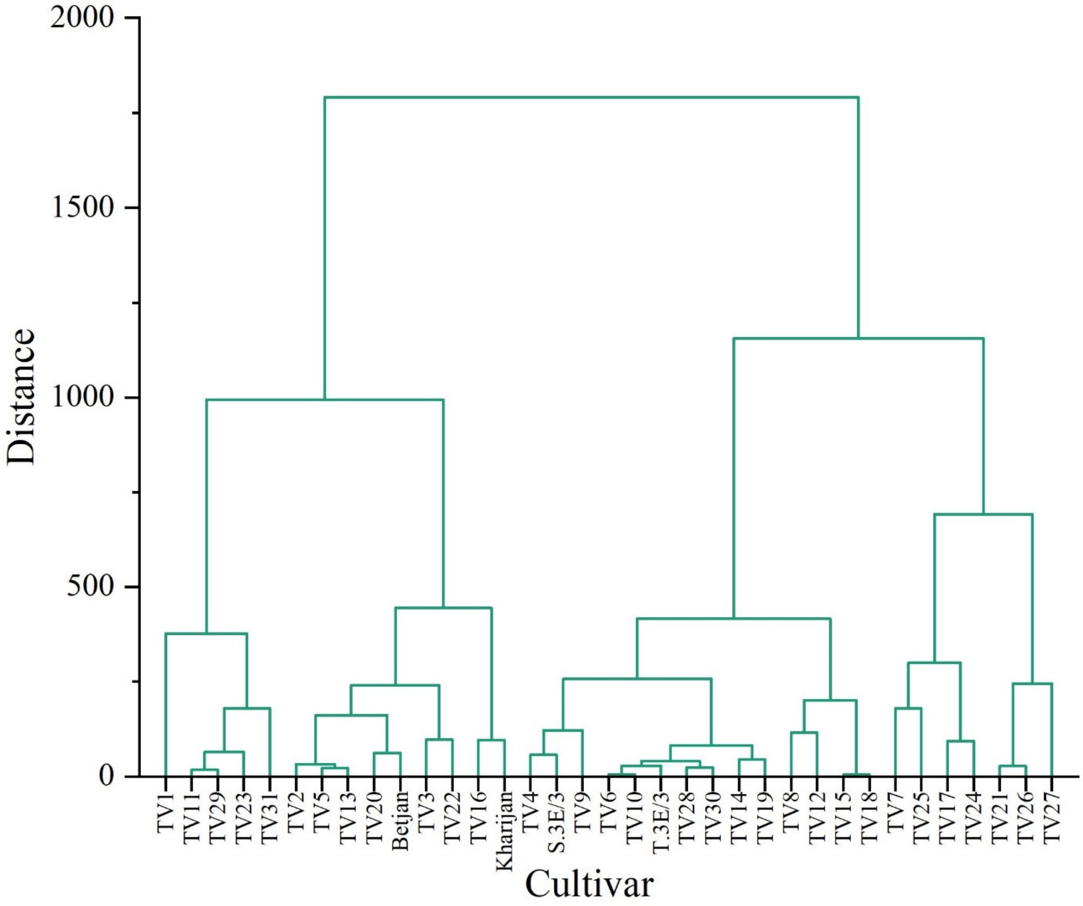

Figure S9. Dendrogram representing clustering of cultivars based on ferric reducing antioxidant potential (FRAP) of cultivars in monsoon.

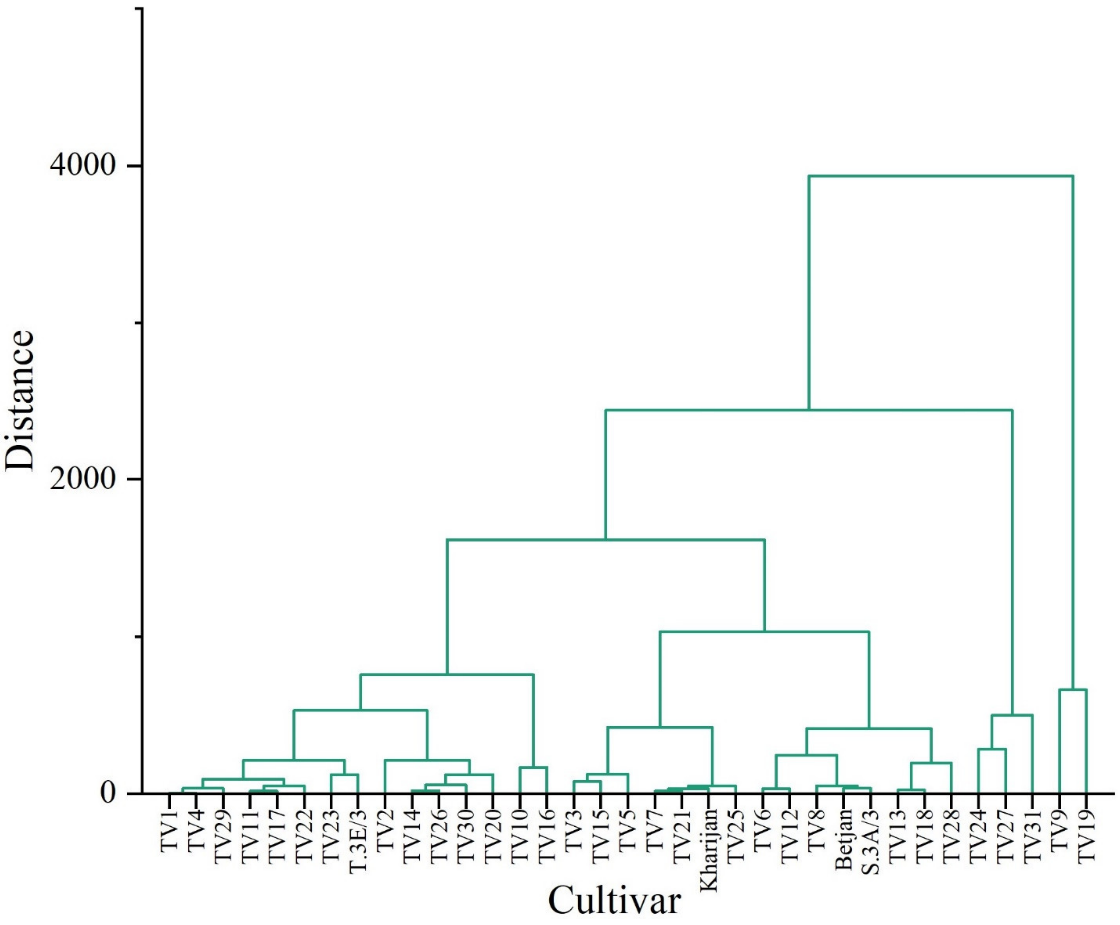

Figure S10. Dendrogram representing clustering of cultivars based on ferric reducing antioxidant potential (FRAP) of cultivars in autumn.

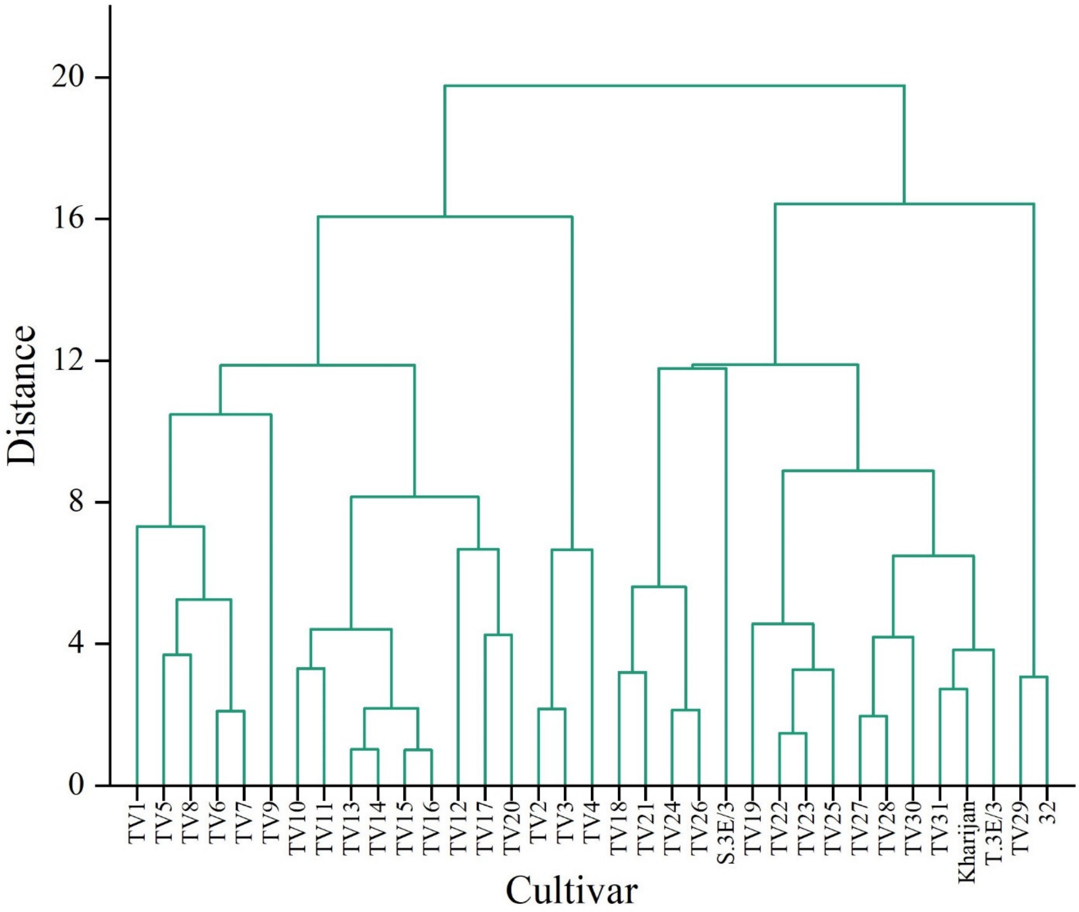

Figure S11. Dendrogram representing clustering of cultivars based on lipid peroxidation inhibition activity of cultivars in pre-monsoon.

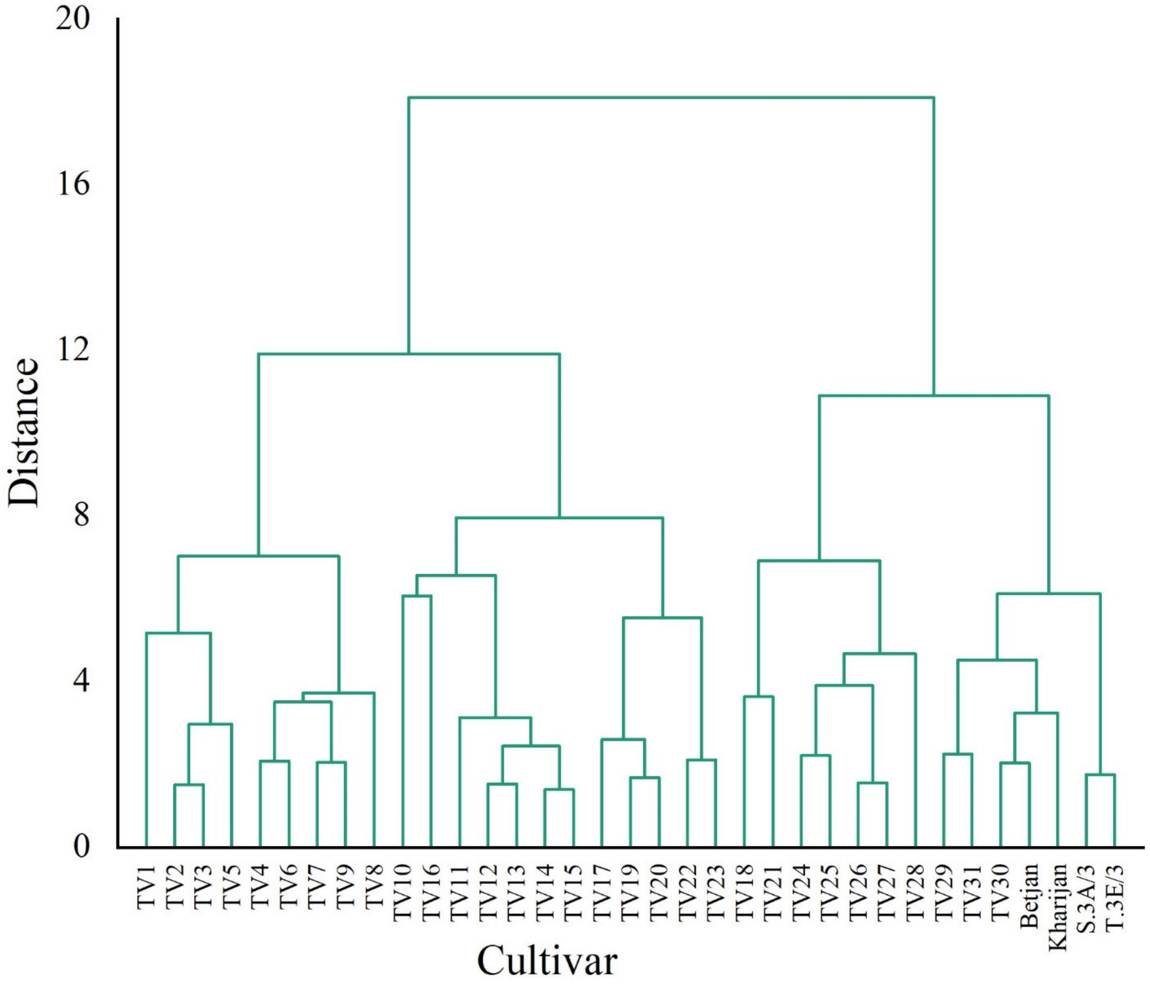

Figure S12. Dendrogram representing clustering of cultivars based on lipid peroxidation inhibition activity of cultivars in monsoon.

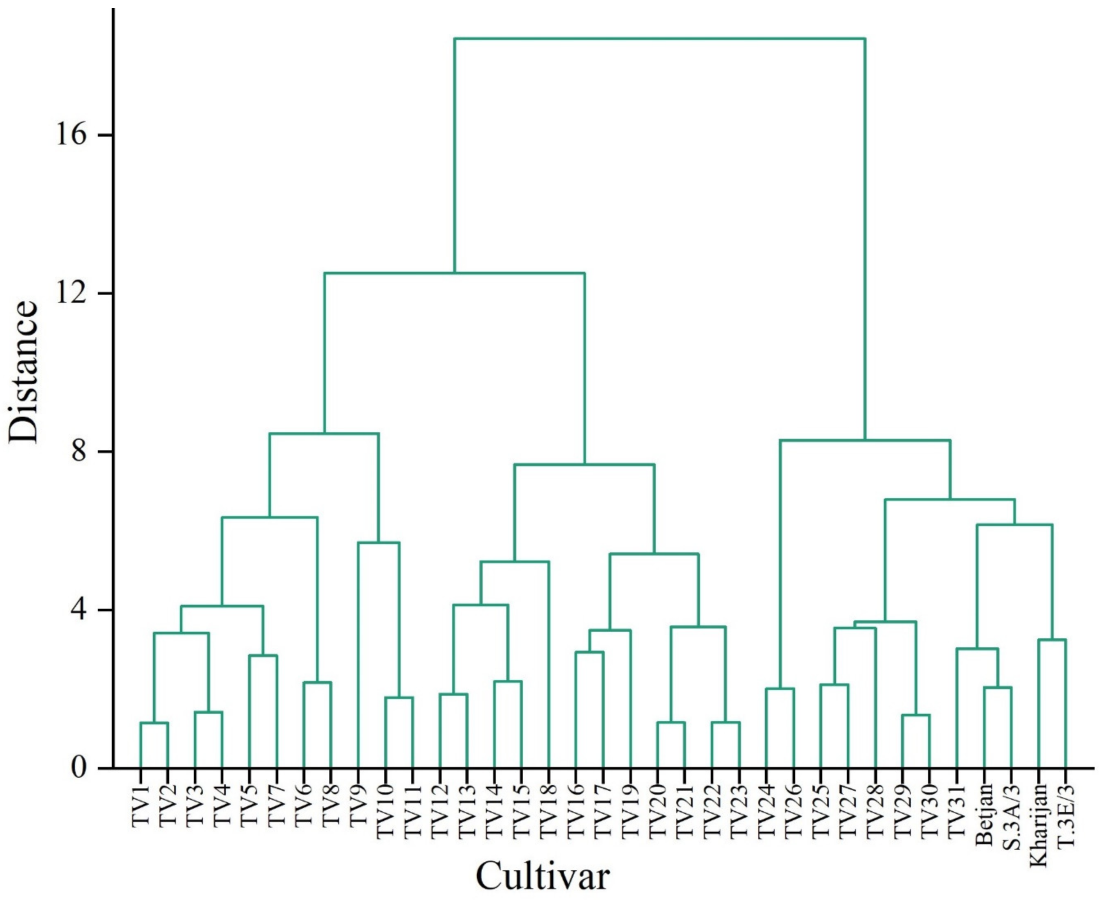

Figure S13. Dendrogram representing clustering of cultivars based on lipid peroxidation inhibition activity of cultivars in autumn.
